# Heel-strike mechanics reveal evolutionary trade-offs in hominin bipedalism

**DOI:** 10.1073/pnas.2533918123

**Published:** 2026-09-08

**Authors:** Nicholas B. Holowka, Matthew C. O’Neill, Vincent Bhandal, Otto Lam, Zacchariah M. Apolito, Caleb A. Massimi, Kevin G. Palmisano, Steven Worthington, Brigitte Demes, Nathan E. Thompson

**Affiliations:** ^a^ Department of Anthropology, The Pennsylvania State University, University Park, PA 16803; ^b^ Huck Institute of Life Sciences, The Pennsylvania State University, University Park, PA 16803; ^c^Department of Anthropology, University at Buffalo, Buffalo, NY 14261; ^d^Department of Anatomy, Midwestern University, Glendale, AZ 85308; ^e^ Department of Anatomical Sciences, Renaissance School of Medicine at Stony Brook University, Stony Brook, NY 11794; ^f^ Icahn School of Medicine at Mount Sinai, New York, NY 10029; ^g^ Institute for Quantitative Social Sciences, Harvard University, Cambridge, MA 02138; ^h^Department of Anatomy, College of Osteopathic Medicine, New York Institute of Technology, Old Westbury, NY 11568

**Keywords:** human evolution, biomechanics, chimpanzees, energetics, functional morphology

## Abstract

The human walking step is initiated by a distinctive heel-strike. Our closest living relatives, the African apes, are among the few other animals that are also thought to heel-strike. Qualitative similarities between human and African ape heel-strikes have been invoked in theories about the origins of hominin bipedalism, but some quantitative data suggest possible interspecies differences in foot-strike mechanics that hint at more complex evolutionary scenarios. However, these data are currently too sparse to fully characterize these differences or understand their functional consequences for hominin evolution. To address these gaps, we collected detailed three-dimensional marker-based kinematic and ground reaction force data in humans and in chimpanzees walking bipedally and quadrupedally. We found that chimpanzees used 2.4 to 8.6 times greater ranges of foot-strike angles than humans and often did not heel-strike. Statistical models revealed a possible explanation for this finding, showing that in both chimpanzees and humans, heel-striking is associated with high impact peak forces and loading rates. We also found that humans expend 26 to 41% more metabolic energy when they contact the ground with the distal foot before the heel, indicating an important adaptive trade-off: Heel-striking lowers the energetic cost of bipedal walking but increases potentially damaging impact loading rates. These results suggest that early hominins with primitive lower limb anatomy faced a choice between high impacts and relatively uneconomical walking, either of which likely constrained their terrestrial mobility. The later evolution of larger heels and lower limb joints enabled safe, economical heel-striking and greater daily ranging.

## Significance

The “heel-strike” that initiates the typical human walking step has been observed in very few species outside our closest living relatives, the African apes, but the evolutionary origin and functional consequences of this unusual gait feature remain both contentious and poorly understood. Here, we present detailed 3-D kinematic and kinetic data from humans and chimpanzees which show that the human heel-strike is biomechanically distinct and thus likely represents an adaptation to bipedalism in hominins. We also present metabolic data which suggest human heel-striking evolved to save energy but came at the cost of exposure to higher impact forces. These results indicate that heel-striking involved adaptive trade-offs for hominin bipedalism that helped shape the anatomy and ecology of our species.

Humans are defined, in part, by our unique form of bipedal locomotion that involves a suite of distinctive characteristics that interact to reduce muscle force, work, and power requirements, thereby allowing us to walk with relatively low energy expenditure (1–4). One unusual and poorly understood aspect of human walking is our use of “heel-strikes,” wherein the heel is the first (and only) part of the foot to contact the ground at the beginning of a walking stride. This foot-strike posture (i.e., first part of the foot to contact the ground) has only been observed in a few quadrupedal mammalian taxa (5, 6), including the other great apes (7–13). The qualitative similarities between human and African ape heel-strikes have been taken as evidence of a terrestrial origin for hominin bipedalism (5, 14, 15) and suggest that walking with a heel-strike is a primitive condition for early hominins (7, 10, 16, 17). However, some quantitative biomechanical data suggest possible differences in human and African ape heel-strikes (2, 9, 18, 19), and several recent fossil hominin discoveries have been interpreted as showing potential use of alternative foot-strike kinematics (20, 21). These different pieces of evidence hint at the possibility that novel heel-strike mechanics evolved within the hominin lineage as an adaptation for bipedality. However, current quantitative data are too sparse to evaluate how humans and nonhuman apes differ in foot-strike mechanics and why such changes would have been selectively advantageous for hominins.

Humans and African apes differ from most other primates in their use of plantigrade foot postures, wherein the whole plantar surface of the foot, including the heel, touches the ground during stance phase of a walking stride (8, 14). Humans always heel-strike when walking (9), and many previous studies have qualitatively described African apes using heel-strikes during quadrupedal walking (7, 8, 10, 12, 22). However, plantar pressure recordings indicate that, unlike humans, bonobos and chimpanzees often exhibit nearly simultaneous heel and midfoot contact when walking, especially during bipedalism (6, 9, 19, 23). Additionally, recent experimental data indicate that during bipedal walking chimpanzees contact the ground with more plantarflexed ankles than humans and consequently experience different ankle joint moments (1, 2, 18, 24). These findings correspond to qualitative descriptions of some kinematic differences between human and African ape heel-strikes (8, 12, 19, 22), but more data are needed to comprehensively quantify and assess the significance of these differences. Further, matching data from both bipedal and quadrupedal walking are necessary to evaluate the effects of adopting bipedalism on early hominin foot-strike mechanics.

If human heel-strikes do differ from those used by African apes, then the differences may provide some selective benefit for bipedal locomotion. Indeed, several mechanical models have been proposed to explain how heel-striking can reduce the metabolic cost of walking in plantigrade species (10, 25–28, see *SI Appendix*, *Discussion*). These models provide a strong theoretical basis for predicting that the capacity to heel-strike evolved in great apes and was later refined in hominins to reduce walking costs (10, 27). However, no data currently exist that directly quantify or verify the energetic benefits of this foot-strike posture during walking.^*^ Apart from this potential benefit, one proposed cost of heel-striking is the high “impact force” that has been observed immediately following foot contact in human walking (*SI Appendix*, Fig. S1) (16, 17, 27, 29, 30). The impact force impulse occurs when the lower limb rapidly decelerates following the heel’s collision with the ground (31), and its high loading rate makes it potentially damaging to joint cartilage (31–35; but see refs. 36 and 37). During running, humans can reduce impact loading rates by contacting the ground with parts of the foot distal to the heel (i.e., metatarsal heads or lateral foot), which increases ankle compliance and thereby decreases lower limb momentum change (38). A similar mechanism could exist during walking, although walking and running mechanics are very different from one another in humans (39). Further, there are currently no published data on impact forces or loading rates during nonhuman ape locomotion, preventing an assessment of whether these forces are uniquely high in humans and would have represented a mechanical cost of bipedal heel-striking in early hominins.

Determining the functional consequences of heel-striking, in addition to quantitatively comparing foot-strike in humans and other apes, is critical to understanding the evolution of hominin bipedalism. Observations of heel-strikes in all extant great ape species have contributed to the notion that heel-striking would have been part of bipedal walking kinematics in the earliest hominins (7, 15, 17), and interpretations of fossil hominin morphology and evolution have generally been assessed under this paradigm (e.g., refs. 40 and 41). However, these interpretations are currently limited by our poor understanding of nonhuman ape foot-strike mechanics, especially during bipedalism. To address these gaps and thereby improve our knowledge of the evolution of hominin locomotion, we collected detailed 3-D kinematic and ground reaction force (GRF) data at foot-strike in humans and in chimpanzees walking both quadrupedally and bipedally. We compared foot-strike kinematics between species and locomotor modes and tested for relationships between heel-striking and impact force magnitudes and loading rates. We also quantified differences in metabolic energy expenditure in humans walking with and without heel-strikes to test the prediction arising from previously developed mechanical models that heel-striking provides energetic benefits (10, 25, 27, 42). This biomechanical and energetic analysis reveals how human heel-strike mechanics are different from those used by chimpanzees and suggests that the capacity to use these mechanics likely evolved within the hominin lineage to confer selective benefits for bipedal walking.

## Results

**Fig. 1.**
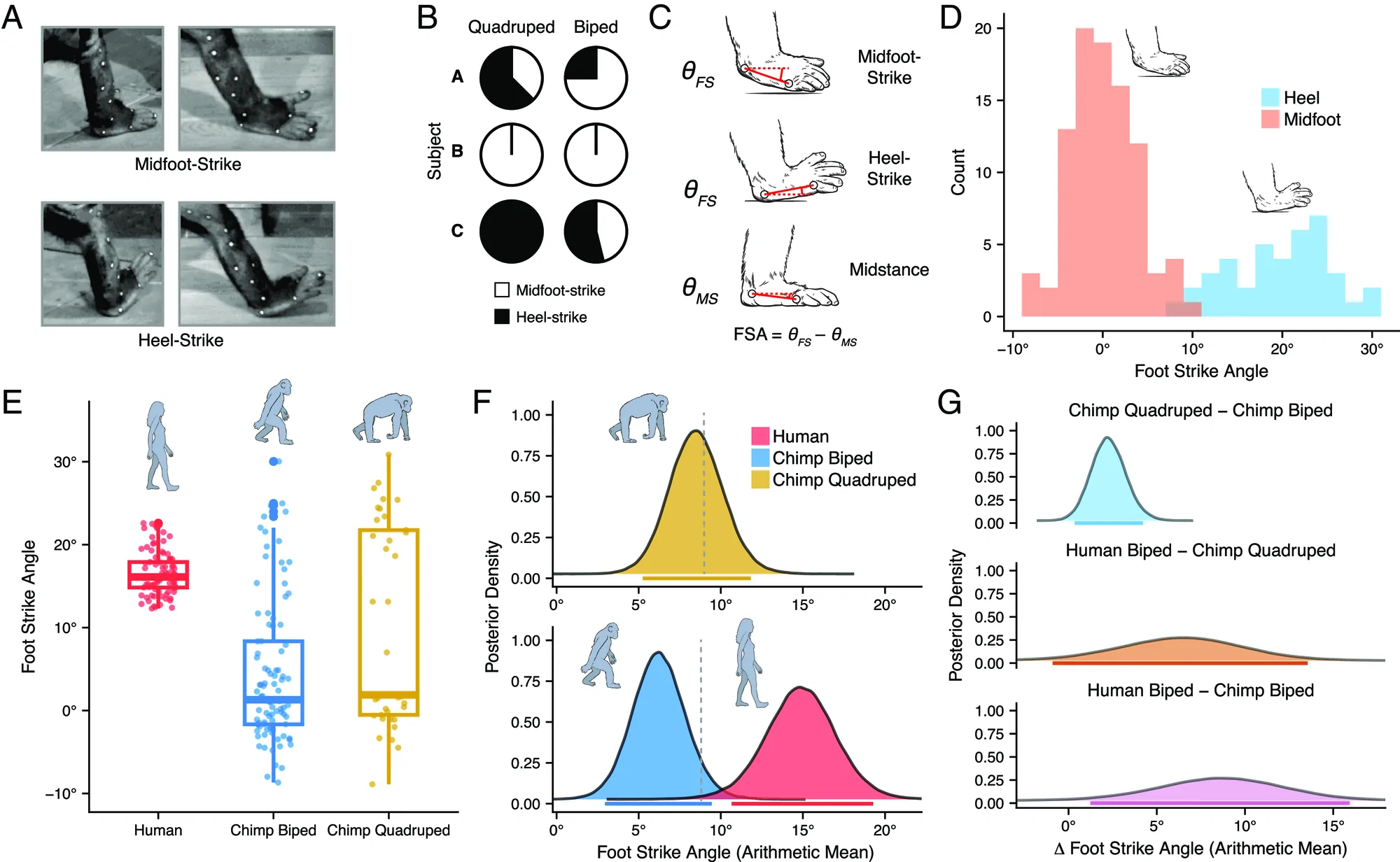
Foot-strike postures used by chimpanzees compared to those used by humans. (*A*) Images from high-speed videos of chimpanzee walking depicting representative foot-strikes. (*B*) Pie-charts representing the proportion of steps coded as midfoot- (white) and heel-strikes (black) for each chimpanzee subject. (*C*) Angles and equation used to calculate foot-strike angle (FSA). (*D*) Histograms representing numbers of steps at each FSA (n = 133, 2° intervals). Colors indicate steps coded as midfoot- (orange) and heel-strikes (blue) based on visual assessment of videos. (*E*) Boxplots depicting raw data values of FSAs for human steps (n = 89), bipedal chimpanzee steps (n = 96), and quadrupedal chimpanzee steps (n = 37). Points represent individual steps, boxes represent interquartile ranges, middle bars represent median values, and whiskers extend to the most extreme data point ± 1.5 times the interquartile range. (*F*) Posterior density plots depicting Gaussian linear model-predicted distributions of FSA values for chimpanzees walking quadrupedally (*Top*), and chimpanzees and humans walking bipedally (*Bottom*). The vertical dashed line indicates approximate threshold between midfoot- and heel-strikes. (*G*) Posterior density plots depicting Gaussian linear model predicted distributions of arithmetic mean differences in FSA for chimpanzee quadrupedalism minus chimpanzee bipedalism (*Top*), human bipedalism minus chimpanzee quadrupedalism (*Middle*), and human bipedalism minus chimpanzee bipedalism (*Bottom*). For (*F* and *G*), solid horizontal lines below distributions indicate 95% highest posterior density intervals (HPDI).

We collected kinematic and GRF data from three male chimpanzees walking on a flat runway (*SI Appendix*, Table S1). We used high-speed, high-resolution videos to qualitatively categorize foot-strike postures as “heel-strikes” or “midfoot-strikes” based on what part of the foot contacted the ground first at the beginning of a stride (Fig. 1*A* and Movies S1–S6). Foot-strike posture was variable within and between subjects; across bipedal and quadrupedal steps, Chimp A only used heel-strikes in 31 out of 76 strides, Chimp B never used heel-strikes (0/43 strides), and Chimp C predominantly used heel-strikes (17/29 strides) (Fig. 1*B* and Movies S1–S6). However, Chimps A and C both used heel-strikes less frequently when bipedal than when quadrupedal. We also calculated foot-strike angle (FSA) as the angle of the foot’s long axis relative to the ground at initial contact (38, 43) using the 3-D coordinates of markers on the foot (Fig. 1*C*). Across subjects the FSA ranges were 39.7° for quadrupedal strides (n = 37, −8.9 to 30.9°) and 38.7° for bipedal strides (n = 96, −8.7 to 30.0°) (Fig. 1 *D* and *E*). Within subjects, FSA ranges were 39.7° for Chimp A, 11.1° for Chimp B, and 23.8° for Chimp C (*SI Appendix*, Fig. S2). This FSA variation was not related to walking speed (*SI Appendix*, Fig. S3) and was only very weakly related to hip and knee joint angles at foot-strike (*SI Appendix*, Fig. S4). All strides but one with FSAs > 9° had been qualitatively coded as heel-strikes, and all strides but one with FSAs ≤ 9° had been coded as midfoot-strikes. This cutoff between foot-strike types is like the cutoff in human running (43), and it corresponds to two distinct clusters in the chimpanzee FSA distribution (Fig. 1*D*), supporting our qualitative categorization of chimpanzee foot-strikes. For comparison, we measured FSA in nine adult humans who walked barefoot across a force-plate instrumented runway (*SI Appendix*, Table S2). All human steps were heel-strikes, and the 10.3° range of all human FSAs (12.3 to 22.6°) was only 26% of the chimpanzee FSA range (Fig. 1*E*) and was less than that used by any single chimpanzee subject in our study (*SI Appendix*, Fig. S2). Individually, chimpanzee FSA ranges were 2.4 to 8.6 times greater than the average FSA range of human subjects [4.6±1.2° (mean ± SD)].

To compare human and chimpanzee foot-strike postures and predict species-level differences we constructed Bayesian Gaussian models that had subject-specific random intercepts and controlled for body mass and walking speed (Fig. 1*F*). Our statistical models predicted that humans have 1.2 to 15.9° higher FSAs than chimpanzees walking bipedally (95% HPDI), and 0.9° lower to 13.6° higher FSAs than chimpanzees walking quadrupedally (95% HPDI) (Fig. 1*G*). The models also predicted that the posterior probabilities (P) that humans have higher FSAs than chimpanzees walking bipedally and quadrupedally were 0.986 and 0.958, respectively. The models predicted that chimpanzees have higher FSAs when walking quadrupedally than bipedally (P = 0.992) and that this difference ranged from 0.3° to 4.2° higher in quadrupedal walking (95% HPDI) (Fig. 1*G*; see *SI Appendix*, Fig. S5 for individual subject contrasts). Finally, the models predicted that chimpanzees have an average bipedal FSA below the 9° threshold associated with midfoot-strikes (P = 0.952), indicating that they frequently midfoot-strike when bipedal. Their average quadrupedal FSA was not clearly below this threshold (P = 0.624), consistent with their more frequent use of heel-strikes when walking quadrupedally (Fig. 1*F*). Thus, the chimpanzees in our study differed from humans in both their more frequent use of midfoot-strikes and their much more variable foot-strike postures.

**Fig. 2.**
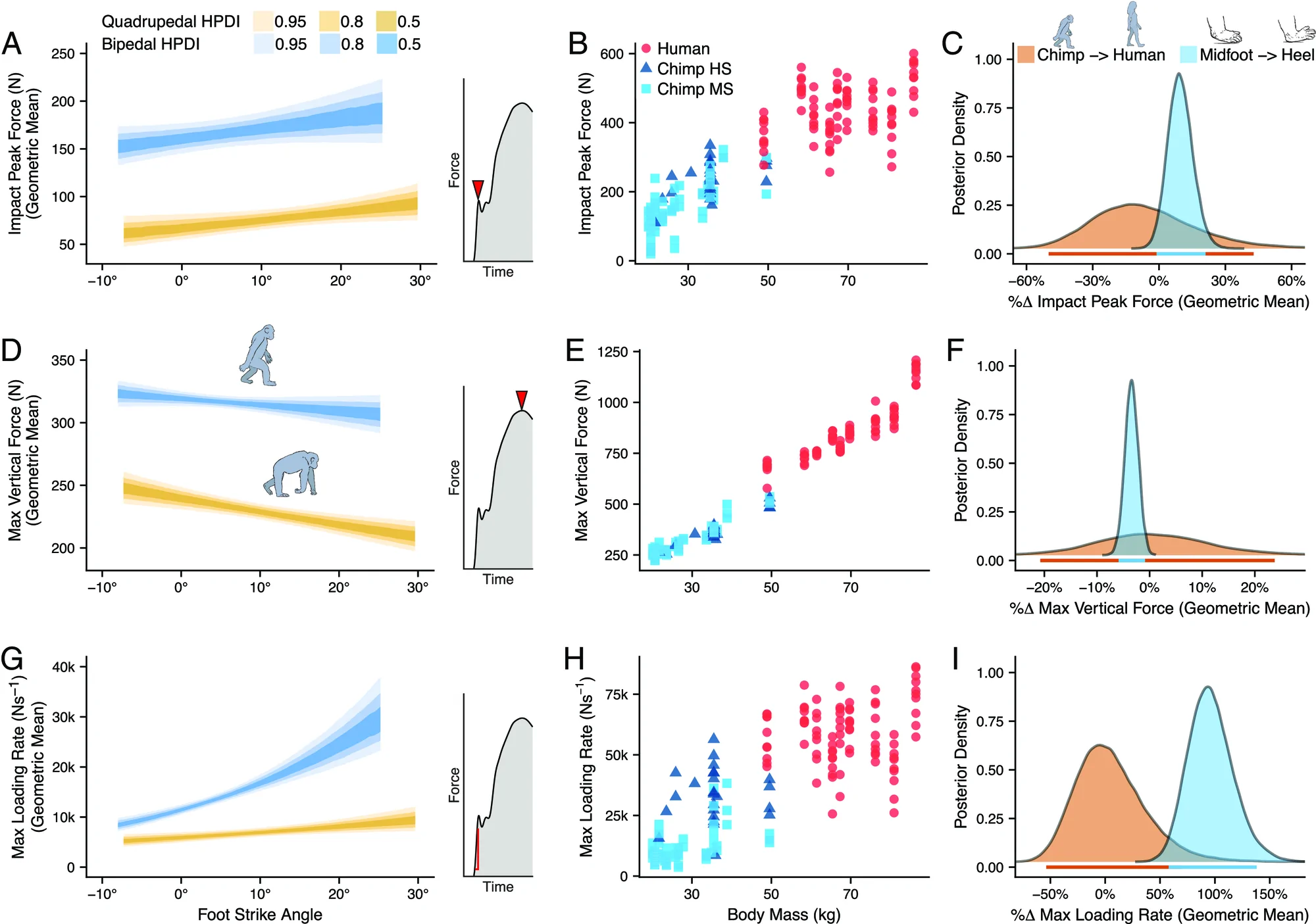
Ground reaction force (GRF) variables and foot-strike postures in chimpanzees and humans. Results depicted for impact peak force (*A*–*C*), max vertical force (*D*–*F*), and max loading rate (*G*–*I*). (*A*, *D*, and *G*) GRF variables versus FSA in chimpanzees walking bipedally (blue) and quadrupedally (yellow). Shaded polygon ribbons represent lognormal linear model-predicted HPDI for each FSA angle, with the ribbons representing the 50%, 80%, and 95% HPDIs from most to least opaque. Images to the right of the graphs depict representative vertical GRF over roughly the first 40% of chimpanzee stance with the arrow (*A* and *D*) or lines (*G*) depicting the force variable. (*B*, *E*, and *H*) Body mass (kg) versus force variables during bipedal walking in humans and chimpanzees. Individual dots represent steps from humans (n = 89) (red circles), chimpanzee midfoot-strikes (n = 76) (light blue squares), and chimpanzee heel-strikes (n = 25) (dark blue triangles). (*C*, *F*, and *I*) Posterior density plots depicting lognormal linear model-predicted distributions of percent difference (∆%) in the geometric mean of each force variable for bipedal chimpanzee heel-strikes vs. midfoot-strikes (blue), and human heel-strikes vs. bipedal chimpanzee heel-strikes (orange). For the former, positive percentages indicate higher values for heel-strikes, and for the latter positive percentages indicate higher values for humans. Solid horizontal lines below plots indicate 95% HPDI.

We used vertical GRF data from the human and chimpanzee strides to calculate three force variables (Fig. 2 *A, D*, and *G* and *SI Appendix*, Fig. S1): impact peak force (IPF), maximum vertical force (MVF), and maximum loading rate (MLR). We could detect distinct impact peaks in GRF data from 74% and 42% of chimpanzee *quadrupedal* heel- and midfoot-strikes, respectively, and from 100% and 76% of chimpanzee *bipedal* heel- and midfoot-strikes, respectively. To assess the relationships between force variables, foot-strike posture, and locomotor mode in chimpanzees we used Bayesian lognormal mixed-effects models that had subject-specific random intercepts and that controlled for body mass and walking speed. We did not divide force variables by body weight as is common in gait biomechanics research (44) as we did not want to assume linear relationships between these variables and body mass. Our statistical models predicted that IPF increases with FSA for both bipedal (P = 0.952) and quadrupedal (P = 0.98) strides and that the rate of increase is a modest −0.2 to 2.7 N deg^−1^ (95% HPDI) for bipedalism, and 0.1 to 1.9 N deg^−1^ (95% HPDI) for quadrupedalism (Fig. 2*A* and *SI Appendix*, Fig. S6 *A* and *B*). Conversely, the models predicted that MVF decreases with FSA for both bipedal (P = 0.914) and quadrupedal (P = 1) strides, but that again, the rates of these decreases are relatively modest: −1.2 to 0.2 N deg^−1^ (95% HPDI) for bipedalism, and −1.7 to −0.5 N deg^−1^ (95% HPDI) for quadrupedalism (Fig. 2*D* and *SI Appendix*, Fig. S6 *C* and *D*). By comparison, the models predicted MLR increases strongly with FSA in bipedal (P = 1) and quadrupedal (P = 0.997) strides, with the 95% HPDIs for rates of increase being 463 to 906 Ns^−1^ deg^−1^ and 34.5 to 226.5 Ns^−1^ deg^−1^ for bipedalism and quadrupedalism, respectively (Fig. 2*G* and *SI Appendix*, Fig. S6 *E* and *F*).

To compare human and chimpanzee GRFs during bipedal walking, as well as foot-strike posture within chimpanzees, we constructed Bayesian lognormal mixed-effects models with subject-specific random intercepts where we controlled for body mass and walking speed. Our statistical models predicted that when bipedal, chimpanzees have modestly higher IPFs (P = 0.963) and lower MVFs (P = 0.997) when heel-striking compared to midfoot-striking. The 95% HPDIs for these differences were 1.1% lower to 21.1% higher IPFs when heel-striking, and 0.9 to 5.9% higher MVFs when midfoot-striking (Fig. 2 *C* and *F* and *SI Appendix*, Fig. S7). The models also predicted much higher MLRs for bipedal chimpanzees when heel-striking versus midfoot-striking (P = 1). The 95% HPDI of this difference was 57.9 to 138.1% higher MLRs when heel-striking (Fig. 2*I* and *SI Appendix*, Fig. S7). The models predicted wide 95% HPDIs for differences between human and bipedal chimpanzee heel-strikes for all three force variables: 50% lower to 42.8% higher IPFs in humans (Fig. 2 *B* and *C*), 20.8% lower to 23.8% higher MVFs in humans (Fig. 2 *E* and *F*), and 50.3% lower to 70.1% higher MLRs in humans (Fig. 2 *H* and *I*). However, for each variable the predicted difference between species was likely to be less than 25% (IPF: P = 0.688; MVF: P = 0.965; MLR: P = 0.595; all three HPDIs encompassed 0). Thus, we did not find evidence of large differences in these GRF variables between humans and chimpanzees when both walk bipedally with heel-strikes.

**Fig. 3.**
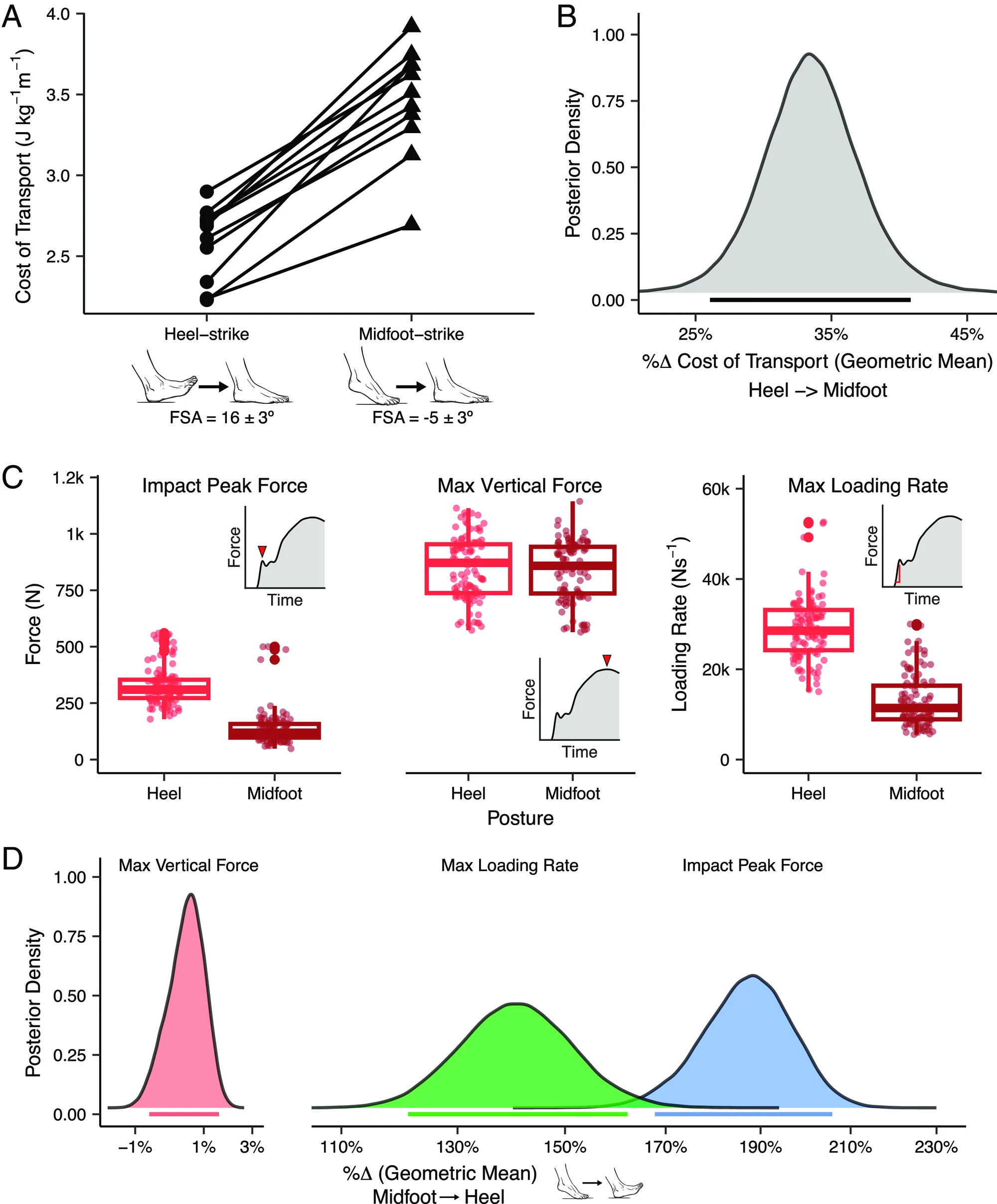
Net metabolic cost of transport (COT) and ground reaction force (GRF) variables in human heel-strike versus midfoot-strike walking. (*A*) COT in heel-strike (circles) and midfoot-strike (triangle) walking for each human participant (n = 11) at a speed corresponding to a Froude number of 0.18. Mean ± SD of FSA for all participants for both postures indicated below graph. (*B*) Posterior density plot depicting lognormal linear model-predicted distribution of percent change (∆%) in the geometric mean of COT from heel-strike to midfoot-strike, with higher percentages indicating higher values for midfoot-strikes. (*C*) Boxplots depicting impact peak force (IPF), maximum vertical force (MVF), and maximum loading rate (MLR) raw data values for humans walking with heel-strikes (n = 110) and midfoot-strikes (n = 110). Points represent individual steps, boxes represent interquartile ranges, middle bars represent median values, and whiskers extend to the most extreme data point ± 1.5 times the interquartile range. Inset images depict representative vertical GRF over roughly the first 40% of stance with the arrow or lines depicting the force variable. (*D*) Posterior density plots depicting the lognormal linear model-predicted distributions of percent change (∆%) in the geometric means of IPF (blue), MVF (pink), and MLR (green) from midfoot-strike to heel-strike in human walking, with higher percentages indicating higher values for heel-strikes. For (*B* and *D*), solid horizontal lines below distributions indicate 95% HPDI.

Last, we investigated the effects of foot-strike posture on metabolic energy expenditure and impact forces in 11 human subjects (*SI Appendix*, Table S2) walking on a treadmill and a force plate-instrumented runway using two different foot-strike types: heel-strikes (FSA = 16.2 ± 2.6°) and midfoot-strikes (FSA = −4.6 ± 3.4°) (Fig. 3*A* and Movies S7 and S8). When walking with heel-strikes, subjects had average net metabolic costs of transport (COT) of 2.59 ± 0.23 J kg^−1^m^−1^, versus 3.46 ± 0.34 J kg^−1^m^−1^ when walking with midfoot-strikes (Fig. 3*A*). We modeled the relationship between COT and foot-strike postures and predicted that humans have a higher COT when walking with midfoot-strikes than when walking with heel-strikes (P = 1) and that the 95% HPDI of this difference was 26.1 to 40.8% higher when midfoot-striking (Fig. 3*B*). To test for differences in GRF variables between human heel- and midfoot-striking, we constructed Bayesian lognormal mixed-effects models with subject-specific random intercepts controlling for body mass and walking speed. These statistical models predicted that humans have only 0.6% lower to 1.5% higher MVFs when heel-striking compared to midfoot-striking (Fig. 3 *C* and *D*). In contrast, humans have 168 to 206% higher IPFs (95% HPDI) and 121 to 162% higher MLRs (95% HPDI) when heel-striking (Fig. 3 *C* and *D*).

## Discussion

Our results reveal marked differences in human and chimpanzee foot-strike mechanics that suggest hominins evolved specialized anatomy to walk bipedally with economical, high-impact heel-strikes. Unlike humans, the chimpanzees in our study all exhibited highly variable FSAs and regularly made initial ground contact with parts of the foot distal to the heel (i.e., midfoot-strikes). This difference from humans cannot be explained by overall lower limb extension, as the statistical associations between FSA and hip and knee joint angle in chimpanzees were very weak (*SI Appendix*, Fig. S4). Differences in limb length are also unlikely to be a factor, as humans consistently heel-strike by the time they are two years old (45) despite having relatively short limbs. Further, our results demonstrate that chimpanzees that are capable of walking with heel-strikes often do not. Thus, the human heel-strike is not simply a by-product of other aspects of walking kinematics or lower limb anatomy. Our results come from three chimpanzees that varied considerably in foot-strike kinematics, so to further evaluate their generalizability across a broader range of ages and body sizes, we analyzed marker-based kinematic data from three additional chimpanzees that were collected by other researchers in a separate study (24, 46) (*SI Appendix*, Table S1). We found that those subjects also exhibited a wide FSA range during bipedal and quadrupedal walking (0.2 to 21.3°), with most steps (9/13) under the 9° FSA threshold associated with midfoot-strikes (*SI Appendix*, Fig. S8). We also reviewed two other studies with data from a combined 15 chimpanzees that indirectly support our results by showing that chimpanzees use variable ankle joint postures at foot-strike during quadrupedal walking (10, 47). Our data showed a moderate to strong correlation (r = −0.51 to −0.75, 95% HPDI) between touchdown ankle angle and FSA in chimpanzees (*SI Appendix*, Fig. S9). Laboratory (10) and zoo (47) studies of chimpanzees found touchdown ankle angle ranges of 95 to 143° and 83 to 115°, respectively, which overlap with the 89 to 131° range for the chimpanzees in our study, and are far greater than the 82 to 96° range of our human subjects (*SI Appendix*, Fig. S9*B*). Thus, the relatively variable ankle postures used by chimpanzees in these studies likely correspond to more variable foot-strikes than in humans, supporting our results and indicating that specialized heel-striking evolved within the hominin lineage.

These differences between human and chimpanzee foot-strike kinematics suggest the possibility of a selective advantage for heel-striking in hominins. Previous studies have demonstrated that heel-striking in plantigrade species like humans (25, 27) and African apes (10) effectively lengthens the limb by allowing greater anterior translation of the center of pressure under the foot during the first double-support period. These studies predict that heel-striking should reduce the metabolic cost of walking, and here, we provide metabolic data supporting these predictions. We estimated a 26 to 41% increase in net cost of transport when humans switch from heel- to midfoot-striking. The magnitude of this increase is notable, as it occurred in our subjects with minimal changes to their overall walking kinematics outside of the foot (*SI Appendix*, Fig. S10 and Table S3), and is roughly six times greater than the increase observed in a previous study when humans switched from heel- to midfoot-striking when running (48) (*SI Appendix*, Table S4). It is also considerably greater than the cost increases observed in other experimental studies where human subjects altered their normal walking kinematics, including doubling their step widths (49), increasing and decreasing their step frequencies by 15% (50), and walking without arm swing (51) (*SI Appendix*, Table S4). Because humans are adapted for specialized heel-striking, it is unlikely that chimpanzees or other species that are not would have as large metabolic cost differentials between foot-strike postures. Nevertheless, these metabolic data provide confirmation of previously established models that predict that plantigrade animals should save some energy by heel-striking (*SI Appendix*, *Discussion*). In addition to increasing effective limb length, heel-striking could reduce the energy cost of plantigrade walking by altering muscle activation patterns and/or replacing negative muscle work with passive heel pad energy dissipation (2, 52–55). Future research should determine the relative contributions of these different mechanisms to walking costs.

Despite these possible advantages of heel-striking, the chimpanzees in our study consistently used lower FSAs and more frequent midfoot-strikes when walking bipedally than they did when walking quadrupedally. These results suggest that chimpanzees may be more likely to avoid heel-striking when bipedal. This inference is based on data from three individuals, so future research with comparable experimental data from additional chimpanzees should investigate it further. Nevertheless, the results supporting this inference were consistent across our chimpanzee subjects (*SI Appendix*, Figs. S2 and S5), as well as with plantar pressure data from chimpanzees’ sister species, bonobos (*Pan paniscus*) (9). Those data, from seven individuals, indicate that bonobos make initial contact with parts of the foot distal to the heel more frequently during bipedalism than during quadrupedalism. Thus, they provide independent evidence that *Pan* species are more likely to avoid heel-striking when bipedal, supporting our results and further reinforcing their generalizability.

Chimpanzees may be inclined to avoid heel-striking when bipedal because of the associated high impact forces and loading rates. We found that both force variables are higher in chimpanzee bipedalism than in quadrupedalism and that both are higher when chimpanzees heel-strike. Loading rates increase exponentially with greater FSAs, such that they are 92 to 227% higher when chimpanzees heel-strike during bipedalism than during quadrupedalism (*SI Appendix*, Fig. S11). These results were consistent across the chimpanzees in this study and we observed similar effects of foot-strike posture on impact-related forces in our human subjects, as did a previous human study (27). Computational simulations of bipedal macaque walking also predicted heel-strikes would result in higher impact forces (56), demonstrating cross-study and cross-species agreement with our results. Experimental animal studies have revealed that high loading rates have the potential to damage joint articular cartilage (57–59), and so avoiding heel-strikes when bipedal could reflect a subconscious strategy to minimize tissue damage. This inference is supported by the results of this study and plantar pressure data from bonobos (9) as well as qualitative observations of orangutans (13) that indicate great apes heel-strike less frequently when bipedal than when quadrupedal. Humans have relatively larger lower limb joints than other apes (60), which provides an anatomical means to more widely distribute the high joint reaction forces experienced during bipedal heel-strikes and thereby protect joint tissues. The relatively wide tubers and prominent lateral plantar processes of human calcanei may also help distribute these forces within the calcaneus and thereby reduce the risk of calcaneal fracture due to heel-strike (16, 17, 29).

**Fig. 4.**
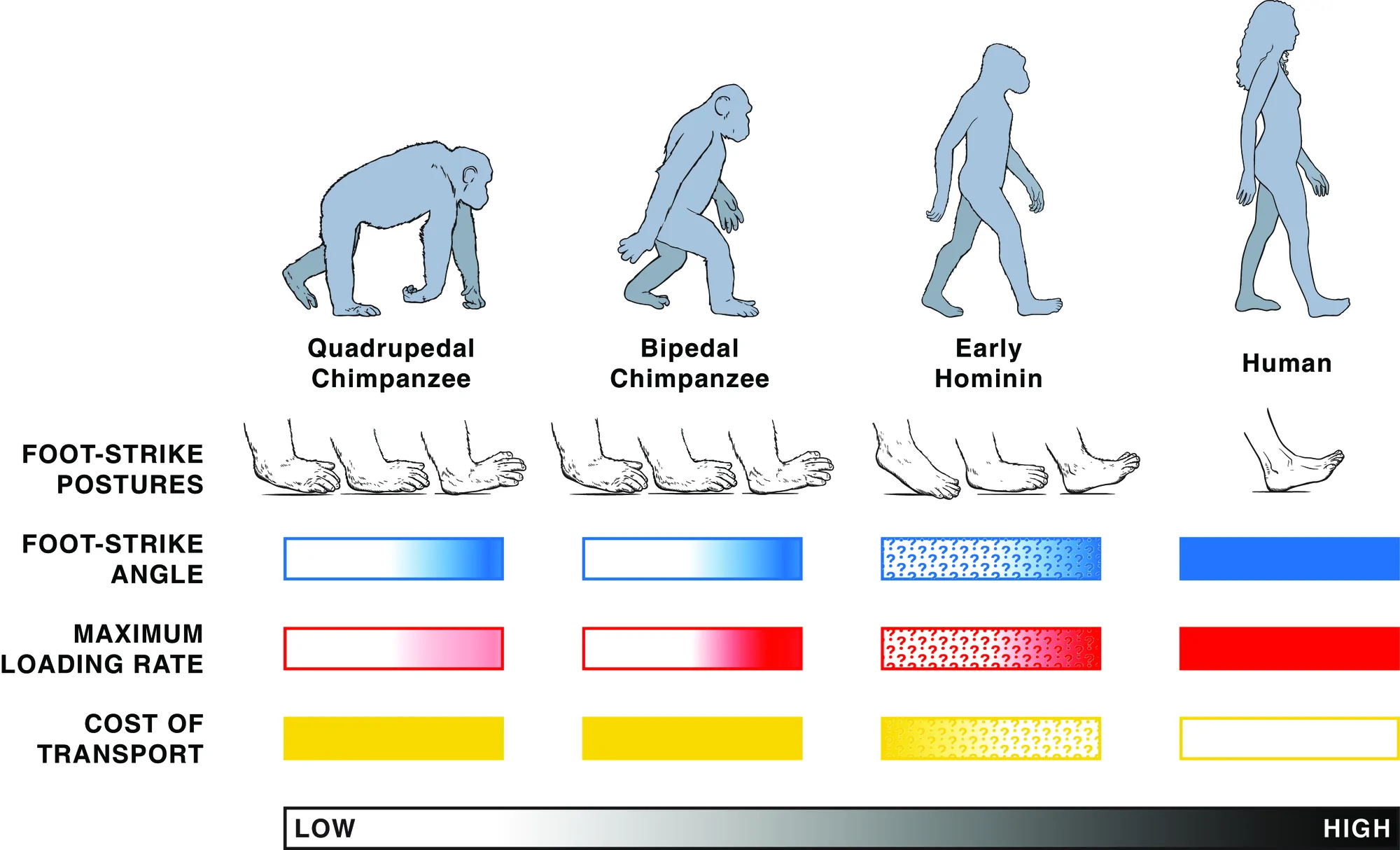
Effects of foot-strike postures on loading rates and walking costs in chimpanzees, humans, and early hominins (non-*Homo* hominin species from the late Miocene and the Pliocene). Color gradients in bars indicate magnitudes of variables, with darker shades indicating higher values. Chimpanzees use a range of foot-strike postures and foot-strike angles during bipedal and quadrupedal walking and thus have a range of maximum loading rates, although loading rates are generally lower during quadrupedalism. Humans only heel-strike and thus have high loading rates but relatively low metabolic costs of transport. Early hominin foot-strike postures are unknown, but these species could have used a range of postures like bipedal chimpanzees, and if so, could have faced a trade-off between loading rates and walking costs that could have led them to limit their daily travel distances.

These morphological traits, when considered alongside the results of this study, let us expand on previously articulated hypotheses about the evolution of heel-striking in hominins (16, 27). The use of heel-strikes distinguishes extant great apes from all other anthropoid primates (7, 8, 11), although observational and morphological data suggest that the frequency and mechanics of these heel-strikes vary between species. Specifically, African apes possess more robust calcanei than orangutans, suggesting that the hominine last common ancestor was better adapted for heel-striking, possibly for greater terrestriality (17). Thus, like chimpanzees, the panin–hominin last common ancestor likely walked quadrupedally with heel-strikes and midfoot-strikes and experienced low but variable impact forces. In adopting bipedalism, the earliest hominins would have experienced substantially higher impact forces and loading rates when heel-striking than their quadrupedal ancestors (Fig. 4). Morphological ancestral state reconstructions suggest that these hominins had African ape-like calcanei (17) and lower limb joint sizes (61) that would have been poorly adapted to withstand the high forces of bipedal heel-strikes. Repeated high-impact, high-rate forces could have gradually damaged lower limb tissues in these small-jointed hominins, which would have limited their mobility over time and perhaps reduced their fitness.

Early hominins could have coped with the high loading rates of heel-strikes in either of two ways (Fig. 4): First, they could have walked bipedally with more frequent midfoot-strikes (like chimpanzees and bonobos), which would have reduced impact-related forces but made locomotion more metabolically expensive, potentially contributing to limits on daily travel (27). Alternatively, they could have used heel-strikes for more economical locomotion but maintained small enough day ranges to avoid impact-related tissue damage (16). Both scenarios suggest that foot-strike mechanics and energetics could have added constraints to daily travel in late Miocene—early Pliocene hominins. Fossil evidence indicates that some mid-Pliocene hominins, like *Australopithecus afarensis*, possessed joint and calcaneus morphology that would have protected against the high forces of heel-striking (16, 29, 61), whereas some later species, like *Australopithecus africanus* and *Australopithecus sediba*, did not (16, 61–63). These morphological differences thus suggest that Pliocene hominin species may have had divergent ecologies characterized by different amounts of terrestrial travel (16) and potentially different foot-strike mechanics (20, 27). Fossil hominin footprints, like those found at Laetoli (64–66), may provide additional clues about foot-strike in these species, but recent studies indicate that the relationship between footprint morphology and gait is complex (41, 67, 68). Therefore, careful experimental research is necessary to determine whether footprints can be used to help reconstruct hominin foot-strike evolution.

The late Pliocene–early Pleistocene postcranial fossil record is currently too sparse to determine exactly when adaptations for safe bipedal heel-striking became fixed in our lineage. However, these adaptations were likely in place by the appearance of *Homo erectus*, whose rapid migration from Africa to southeast Asia after 2 Ma attests to some derived long-distance walking capabilities (69), which would have been abetted by economical heel-strike mechanics. The energy saved by heel-striking would have also benefitted the advent of foraging behaviors associated with hunting and gathering, which require long daily travel distances (70, 71). The potentially damaging effects of high loading rates from heel-striking were likely mitigated by relatively large lower limb joints, which become consistent in hominin fossils following the appearance of *H. erectus* (61, 72). In modern humans, heel-strikes with high impact forces and loading rates are normal features of walking, even in habitually barefoot people (73). There is little evidence that such forces damage healthy musculoskeletal tissues in modern humans (36, 37), and they may even have anabolic effects on lower limb joint cartilage growth and maintenance (74–78). Thus, evolution has made bipedal heel-striking both safe and economical for humans, enabling it to become a defining feature of our species’ distinctive walking abilities.

## Materials and Methods

### Research Subjects.

We collected experimental walking data from three male chimpanzees over 4 y, during which time they ranged in age from 4.7 to 8.8 y, and in body mass from 20.1 to 49.6 kg (*SI Appendix*, Table S1 and *Supplemental Data*). The chimpanzees had been trained to walk bipedally for 1 h/d, 3 to 5 d/wk for at least 6 mo before the start of data collection. The Stony Brook University Institutional Animal Care and Use Committee approved all research procedures involving chimpanzees prior to the start of data collection. We collected experimental walking data from 12 human subjects (six females, six males; 26.9 ± 5 y, 73.4 ± 14 kg; *SI Appendix*, Table S2), all of whom reported no recent musculoskeletal injury, pain, or neuromuscular condition that would affect walking. The University at Buffalo Institutional Review Board approved all research procedures involving human subjects, and each subject provided written informed consent prior to data collection.

### Kinetic and Kinematic Data Collection.

Chimpanzee subjects walked bipedally and quadrupedally at self-selected speeds on a flat, rigid 11-meter-long runway instrumented with four ground-mounted force plates (AMTI BP400600, Watertown, MA) sampling at 1500 Hz. Subjects followed an animal trainer who provided positive reinforcement via food rewards. Prior to experiments, nontoxic, water-soluble white paint was applied to specific locations on the skin overlying anatomical landmarks that were used as marker positions for lower limb kinematics measurements (18) (Fig. 1 and *SI Appendix*, Figs. S4 and S9). We video-recorded chimpanzee subjects using a high-speed four camera motion capture system (Xcitex, Inc., Woburn MA) that sampled at 150 Hz (Movies S1–S6). We completed initial force plate and kinematic data processing using ProAnalyst v.1.7 (Xcitex, Inc.). We only analyzed strides from the left feet of Chimps A and B because we only placed markers on their left sides. We placed markers on both sides of Chimp C and therefore analyzed strides from both right and left feet.

We recorded nine of the human subjects (five females, four males; 27 ± 5.8 y, 66.7 ± 11.4 kg; *SI Appendix*, Table S2) walking at self-selected speeds on a flat, rigid 7.8-meter-long runway instrumented with three ground-mounted force plates (AMTI BP400600 and BMS400600-2K) that sampled at 2,250 Hz. Prior to experiments, we placed reflective markers on anatomical landmarks overlying the skin of the legs and pelvis following (79). We recorded subjects using an eight camera optical motion capture system (Vicon Motion Systems Ltd., Oxford, UK) that sampled at 150 Hz. Subjects walked using both their normal heel-strikes as well as a midfoot-strike posture. For the latter, subjects were instructed to make initial contact with the ground with the lateral side of the front of the foot near the fifth metatarsal head before bringing the heel down into contact with the ground (Fig. 3*A* and Movies S7 and S8). Subjects practiced this foot-strike posture for at least 10 min prior to data collection, until they were able to walk this way comfortably. We recorded force plate and kinematic data using Nexus v.2.12 (Vicon Motion Systems Ltd., Oxford, UK).

We analyzed all force plate data using custom-written MATLAB (The MathWorks Inc., Natick, MA) software. We filtered all GRF data using fourth order recursive lowpass Butterworth filters with 80 Hz cutoff frequencies. This frequency was selected to minimize smoothing of the impact peak while removing electrical noise and artifacts caused by force plate resonance. We defined foot contact as occurring when the increase in vertical GRF surpassed 4,000 Ns^−1^. We calculated MLR between foot contact and the first local force maximum as the maximum change in force magnitude between each successive recorded data point, multiplied by the force plate sampling frequency. We also calculated IPF magnitude when impact peaks were present (*SI Appendix*, Fig. S1). We defined impact peaks as occurring when the first local force maximum was followed by a rapid drop and then a subsequent rise in force within the first 100 milliseconds of stance. In steps without clear impact peaks, we calculated IPF as the magnitude of vertical GRF at the average time of impact peak in steps that did have visible peaks following (38) (*SI Appendix*, Fig. S1). Last, we calculated MVF as the greatest GRF value measured across stance phase.

We calculated walking velocity as the horizontal distance traveled by a marker on the pelvis over a full stride, divided by the stride duration. We then used velocity to calculate Froude number (Fr), which is a dimensionless measure of legged locomotion speed, and which can be used to determine dynamically similar speeds in animals of different body sizes (80):

$$\mathrm{Fr}=v^{2}/\mathrm{gl},$$

where v is walking speed, g is the Earth’s gravitational constant (9.81 ms^−2^), and l is limb length. For chimpanzees, we used a “functional” limb length (18), defined as the distance between the greater trochanter and the ground at midstance, because chimpanzees walk with relatively flexed hind limb joints. Humans walk with relatively extended lower limb joints, so for humans we used greater trochanter height during standing to define limb length.

We used marker position data to calculate hip, knee, ankle, and foot-strike angles (*SI Appendix*, Figs. S4 and S9). Hip angle is the angle between a line connecting the anterior superior iliac spine to the greater trochanter (pelvis segment) and a line connecting the greater trochanter to the lateral epicondyle (thigh segment). Knee angle is the angle between the thigh segment and a line connecting the lateral epicondyle and lateral malleolus (leg segment). Ankle angle is the angle between the leg segment and a line connecting a marker on the lateral side of the calcaneus and the lateral side of the 5th metatarsal head (foot segment). We calculated FSA following previously described methods (38, 43) as

$$\text{FSA}=\theta_{\text{FS}}-\theta_{\text{MS}},$$

where θ_FS_ is the angle between the foot segment and the ground at the exact moment of foot contact, and θ_MS_ is the angle between the foot segment and the ground at foot flat (roughly 30% of stance) (Fig. 1*C*). We also used this method to calculate FSA from marker coordinate data from 13 strides from three chimpanzees (2 males, 34.2 to 52.8 kg, 6 to 9 y; 1 female, 61.8 kg, 19 y, *SI Appendix*, Table S1) from a separate study (46), which were provided to us by the authors of that study.

### Metabolic Energy Expenditure Data Collection.

We measured metabolic energy expenditure in all human subjects during heel- and midfoot-strike walking. During these experiments, subjects walked on a treadmill at 0.18 Fr (1.25 ± 0.03 m/s) using heel-strikes and midfoot-strikes (Movies S7 and S8). We measured sagittal plane lower limb joint kinematics using a previously established marker set (79), and FSA using markers placed on the heel and fifth metatarsal head as described above. We measured metabolic energy expenditure using indirect calorimetry with a K5 portable respirometry system (COSMED Srl, Rome, Italy) that records breath-by-breath oxygen consumption and carbon dioxide production. First, we recorded while the participant stood still for 7 min, then we recorded data for 7 min while the participant was walking on the treadmill with each foot-strike posture. The order of these walking trials was randomized. We used data from the last 2 min of collection for each activity to calculate the average metabolic rates and deducted the standing metabolic rate value from the walking values to calculate the net metabolic rate for both foot-strike postures. We then divided these values by walking speed and body mass to calculate the mass-adjusted net cost of transport.

### Statistical Methods.

We fit all models within a Bayesian inferential framework (81, 82). Models of FSA used a Gaussian likelihood with an identity link. Models of the force variables (IPF, MVF, and MLR) and of metabolic cost of transport used lognormal likelihoods, for which effects correspond to changes in geometric means when transformed back to the original response scale. Bayesian inference does not rely on large sample sizes to yield valid estimates of effect size uncertainty and allowed us to estimate not only the magnitude of an effect and its uncertainty but also the probability that the effect exceeds or falls below a specific value. Our data were hierarchical in nature, consisting of multiple step observations for each chimpanzee and human subject. To account for this nested dependence, we used mixed-effects models with subject-specific random intercepts. We included body mass and speed as covariates in our models. Our chimpanzee subjects were growing throughout the time of data collection, so their ages and body masses were almost perfectly correlated (r ≥ 0.98). Therefore, we did not include age as a covariate in our models because it would have been redundant with body mass and produced unstable coefficient estimates owing to their high collinearity. We also modeled subject-specific error variances to accommodate heteroscedasticity in some response variables. To present results for individual subjects, we additionally fit companion models with subject-specific random slopes, allowing the gait effect on FSA and the posture effect on the force variables to differ among chimpanzees. For each effect, we report two quantities of interest: 1) the 95% HPDI, the interval within which the unobserved population effect falls with 95% probability, and 2) the posterior probability (P) that the effect exceeds, falls short of, or lies within a prespecified range.

We specified weakly informative priors based on biological plausibility, informed by visualizing prior predictive simulations (83). We deliberately used weakly informative priors so that the data, rather than the prior, would drive the posterior. We used Student’s t-distributions (df = 3, mean = 0, SD = 2.5) for both mean and SD parameters. All effect size estimates were based on 100,000 post-warm-up posterior draws (after 10,000 warm-up iterations), with every fifth draw retained to reduce autocorrelation. We fit models using 10 Markov chains and confirmed convergence using the Gelman–Rubin statistic (R-hat = 1.0; (84)). Model fit was assessed using posterior predictive checks comparing replicated to observed data (83). All analyses were performed in R v. 4.6.0, using the packages {brms} v. 2.23.0 (85), {tidybayes} v. 3.0.7 (86), {posterior} v. 1.7.0 (87), {marginaleffects} v. 0.32.0 (88), {ggplot2} v. 4.0.3 (89), and {dplyr} v. 1.2.1 (90), with Stan v. 2.38.0 (91) used as a computational backend for Bayesian inference. Replication code and data are available in a Zenodo archival repository (92) (https://zenodo.org/records/21728576).

## Supplementary Material

Appendix 01 (PDF)

Dataset S01 (CSV)

Movie S1. Chimpanzee A walking quadrupedally with heel- and midfoot-strikes.

Movie S2. Chimpanzee A walking bipedally with heel- and midfoot-strikes.

Movie S3. Chimpanzee B walking quadrupedally with midfoot-strikes.

Movie S4. Chimpanzee B walking bipedally with midfoot-strikes.

Movie S5. Chimpanzee C walking quadrupedally with heel-strikes.

Movie S6. Chimpanzee C walking bipedally with heel- and midfoot-strikes.

Movie S7. Human subject walking on treadmill with heel-strikes.

Movie S8. Human subject walking on treadmill with midfoot-strikes.

## Data Availability

Processed data and analysis code data have been deposited in Zenodo (92) (https://zenodo.org/records/21728576). Study data are included in the article and/or supporting information.

## Footnotes

^*^Cunningham et al. (28) measured the metabolic cost of human “digitigrade” or “toe” walking, where humans walked without allowing the heel to contact the ground. This is different from the “midfoot-strike” posture investigated to here, where a part of the foot distal to the heel contacts the ground first but then the heel is brought down to ground such that the foot assumes its normal, fully plantigrade posture.
