# Supplementary material for: Heel-strike mechanics reveal evolutionary trade-offs in hominin bipedalism
Source: Proc Natl Acad Sci U S A. 2026 Sep 8;123(37):e2533918123. doi: 10.1073/pnas.2533918123 (PMC13578795; doi:10.1073/pnas.2533918123)
Supplement: Supplementary file 1 [file pnas.2533918123.sapp.pdf]

PNAS

Supporting Information for

Heel-strike mechanics reveal evolutionary trade-offs in hominin bipedalism.

Nicholas B. Holowka[1,2], Matthew C. O'Neill[3], Vincent Bhandal[4], Otto Lam[4], Zacchariah M. Apolito[1], Caleb A. Massimi[5], Kevin G. Palmisano[1], Steven Worthington[6], Brigitte Demes[4], Nathan E. Thompson[7]

Nicholas B. Holowka
Email: nmh5855@psu.edu

This PDF file includes:

Supporting Information Text
Figures S1 to S11
Tables S1 to S4
Legends for Movies S1 to S8
Legend for Dataset S1
SI References

Other supporting materials for this manuscript include the following:

Movies S1 to S8
Dataset S1

Supporting Information Text

Discussion

Biomechanical models predicting metabolic benefits of heel-striking for terrestrial plantigrade species.

In the early 1990s, Gebo (1, 2) and Meldrum (3) debated the presence of plantigrade foot postures in nonhuman primates and their role in the evolution of bipedalism. Gebo argued that among primates, only humans and African apes are plantigrade with clear heel-contact, whereas Meldrum argued plantigrady with heel-contact was much more common among primate species. Schmitt and Larson (4) clarified this debate by systematically observing foot postures in videos of non-human primates walking. They concluded that while hylobatids and atelids could also be classified as plantigrade, only hominids (great apes) contact the ground heel first in what could be described as a 'heel-strike' posture. One issue left open by this debate was the question of why hominids, and in particular humans, heel-strike. Several studies have been published over the last two decades arguing that heel-striking provides mechanical advantages for terrestrial plantigrade species that reduce their metabolic costs of walking. These studies have proposed mechanical models demonstrating this effect, and several have provided empirical data supporting these models. None, however, have provided metabolic data directly testing their model. We review these models below. Our data do not allow us to determine which of these models best explain the large metabolic cost differences we observe between heel-strike and midfoot-strike walking in humans. However, we suspect that mechanisms described in the models likely have some cumulative effect on the economy of plantigrade locomotion that is reflected in our experimental results.

Effective Limb Length Model. This model was originally developed by Pontzer (5) to explain why human vertical ground reaction forces are lower than predicted by a biomechanical model that uses walking speed, stride frequency, limb excursion, and limb length to estimate the metabolic cost of walking (see the "LiMb" model (6)). In essence, the LiMb model suggests that longer limbs reduce metabolic costs of walking by increasing limb contact time and thereby lowering the volume of muscle that must be activated to resist ground reaction forces. This model assumes that the hindlimb can be modeled as an inverted pendulum, with a pendular head located at the hip joint pivoting about the point of contact between the ground and the foot. In this model, the length of the arm of the pendulum was assumed to be roughly equivalent to anatomical hindlimb length, which was measured as the distance from the hip joint to the ground during standing. Pontzer found that for human subjects, when anatomical hindlimb length was used in his model equation, the model predicted higher than observed vertical ground reaction forces. However, Pontzer noted that humans, unlike most other legged terrestrial animals, contact the ground with the proximal part of their foot and leave the ground with the distal part of their foot. Because of this changing center of contact between the foot and the ground during human walking, Pontzer reasoned that the actual pivot point of the human hindlimb inverted pendulum is located under the walking surface (see Fig. 6 in (5)). He suggested that the distance between this virtual pivot point and the hip joint center better represents the length of the hindlimb inverted pendulum arm than anatomical leg length in humans. Accordingly, he found that when he used this "effective" hindlimb leg length in his model equation, he produced more accurate predictions of vertical ground reaction force. Importantly, these ground reaction forces were lower than predicted when using anatomical limb length, indicating less volume of activated muscle and therefore lower metabolic cost of locomotion.

Following Pontzer's model, Webber and Raichlen (7) adapted the term 'effective limb length' (ELL) to account for the added effect of a moving center of pressure on a hind limb modeled as an inverted pendulum. They devised experiments to test whether heel-striking affects leg contact time and gait mechanics in humans in a manner predicted by Pontzer's model. They had human subjects walk with their normal heel-strikes and 'non-heel-strike plantigrady' (c.f. midfoot-striking in present study), finding that the latter reduced ELL. They calculated Froude number, a measure of dimensionless speed (8), using ELL measurements for both foot-strike postures. They found that humans transitioned to running from walking at similar dimensionless speeds, but at lower absolute

speeds when using non-heel-strike plantigrady. They also estimated the cost of locomotion in both heel-strike and non-heel-strike walking based on ground reaction force and kinematic data using previously developed methods (9). They found these estimated costs to be higher for non-heel-strike plantigrady at similar absolute speeds, but to be similar between foot-strike postures when walking at the same dimensionless speed (calculated based on ELL). From their results, they concluded that humans evolved to walk with heel-strikes to reduce metabolic energy expenditure during bipedal locomotion. However, their conclusions were based on mechanical estimates of cost reductions, rather than direct measurements of reduced metabolic costs. Despite this lack of supporting metabolic data, this model has been invoked to explain the African ape and human use of heel-strikes in multiple publications (10–14).

Hip Translation Model. Zeininger et al. (13) adapted the concept of the "effective limb length" to explain how heel-striking could allow for greater center of mass travel distance per step in plantigrade species. As in the studies described above (5, 7), they suggest that contacting the ground heel-first and then pushing-off with the toes involves translation of the center of pressure under the foot, resulting in a virtual pivot point for the body's center of mass that is below the walking surface and thus a lengthened inverted pendulum arm. Unlike the *Effective Limb Length Model* described above, Zeininger et al. suggest that the primary benefit of the longer effective limb length in heel-striking is that it increases the distance that the hip joint translates over the course of a step. Greater step lengths, they argue, should result in lower stride frequencies, which are associated with lower metabolic costs of locomotion in interspecies comparisons (15). Zeininger et al. found evidence to support their model by showing that chimpanzees and gorillas, which they describe as heel-strikers, have longer hip translations than would be predicted based on their anatomical limb length. They suggest that heel-striking thus enables plantigrade apes with relatively short hind limbs to achieve more economical locomotion than they would be able to with non-heel-strike walking postures. Their data support a relationship between heel-striking and a hypothesized mechanical determinant of walking costs (stride frequency), but their data did not allow them to quantitatively evaluate actual foot-strike posture, nor did they have metabolic data to demonstrate an energetic benefit of increased hip translation.

Rolling Foot Model. This model is based on the pioneering robotics research of McGeer (16), who designed what he called a "passive walking" machine capable of walking down a slightly sloped surface on two crutch-like pairs of limbs without a motor or active control. This model included a semi-circular foot with a radius 30% of total limb length that effectively enabled the machine to roll over the planted limb like the spoke of a wheel rolling over the wheel rim. He analogized this rollover motion to the movement of the center of pressure under the human foot when landing on the heel and pushing off from the toes, and later research with human subjects confirmed that the human foot behaves in this manner during walking (17). Adamczyk et al. (18) proposed a mechanical model to explain how this rolling foot could reduce the metabolic cost of human walking by reducing the mechanical work associated with step-to-step transitions (see also (19–21)). Based on an inverted pendulum model of human walking, they hypothesized that a rolling foot with a heel-strike reduces the distance between the legs during the step-to-step transition period, which effectively reduces the necessary change in center of mass velocity trajectory. Reducing this change should also reduce the mechanical work necessary to overcome collisional energy losses and thereby reduce metabolic costs of walking (22). To test their model, they conducted experiments in which human participants walked in boots with arcs of fixed length and radius attached to the bottom (18, 23). They found that arcs around 30% of leg length minimized mechanical and metabolic costs of walking, but that the magnitude of arc radius was relatively unimportant as long as the arc was at least slightly rounded (23).

Importantly, Adamczyk et al. (18, 23) did not explicitly model or test the effects of heel-striking on walking costs. However, their experimental evidence that foot length, rather than radius, predicts the mechanical and metabolic costs of walking with a rolling foot, indirectly supports the mechanical significance of heel-striking. Based on their model, humans must land on their heels to achieve the optimal distance traveled by the center of pressure under the rolling foot. According to their model, a midfoot-strike might not achieve this optimum distance and therefore would not minimize

mechanical work during step-to-step transitions. However, they did not test the effect of midfoot-strikes on rolling foot length, and thus it is unclear whether heel-striking is necessary to achieve the metabolic energy savings predicted by their model.

Energy Dissipation Model. Several studies have suggested that heel-striking could help reduce the metabolic cost of walking in humans by enabling more passive rather than active energy dissipation in early stance phase. Zelik and colleagues (24, 25) quantified the disparity between joint and center of mass-level estimations of mechanical work, and based on their analysis suggested that soft tissue structures perform more than 50% of the negative work associated with collision in human walking through passive deformation (see also (26)). They estimated that these tissues perform roughly 7.5 J of negative work per step (in subjects who were 73 kg on average) and suggested that the heel pad could be a major site of this work. The human heel pad is a specialized structure consisting of elastic adipose tissue in fibrous tissue septa that are anchored to the calcaneus and the skin (27). Numerous in vivo and in vitro loading studies have demonstrated that the human heel pad is capable of dissipating energy in high impact collisions (e.g., 27–29).

Recent experimental evidence from Baines et al. (31) suggests that heel pad deformation accounts for roughly 3.8 J of negative work per step in human walking (in subjects who were 76 kg on average), meaning that it could account for as much as 50% of the passive negative work performed by human soft tissues following a leading limb collision. No study has quantified heel pad deformations or negative work in midfoot-strike walking, but we expect that they would be somewhat lower than those observed in heel-striking. Without heel pad deformation, muscles might have to actively perform this negative work. By rough approximation, this could increase the metabolic cost of walking by 2–3% when individuals walk with midfoot-strikes. This slight increase in energy expenditure is unlikely to fully explain humans' use of heel-strikes by itself, but it could have a cumulative effect on the energetics of heel-strike walking when combined with the effects of the other mechanisms described above.

Somewhat relatedly, Usherwood et al. (32) suggested that heel-striking might allow for safer energy dissipation following foot contact in bipedal walking than non-heel-striking. They argued that high collisional energy dissipation is a necessary evil of the metabolic advantages conferred by inverted pendulum gaits and suggested that by heel-striking, humans can actively dissipate energy to protect more brittle tissues (e.g., bone). Specifically, they argued that heel-striking enables the tibialis anterior to dissipate energy by performing negative work following foot contact, and that the human tibialis anterior is specially adapted for this function. While this mechanism does not directly improve the economy of walking, they suggest that it allows for an overall more efficient use of inverted pendulum dynamics. Their hypothesis is indirectly supported by evidence that humans have relatively larger tibialis anterior muscles (33–36) and longer tibialis anterior moment arms (33, 37–39) than other great apes. Additional electromyographic and mechanical data from nonhuman great apes are needed to further evaluate this hypothesis.

Figures

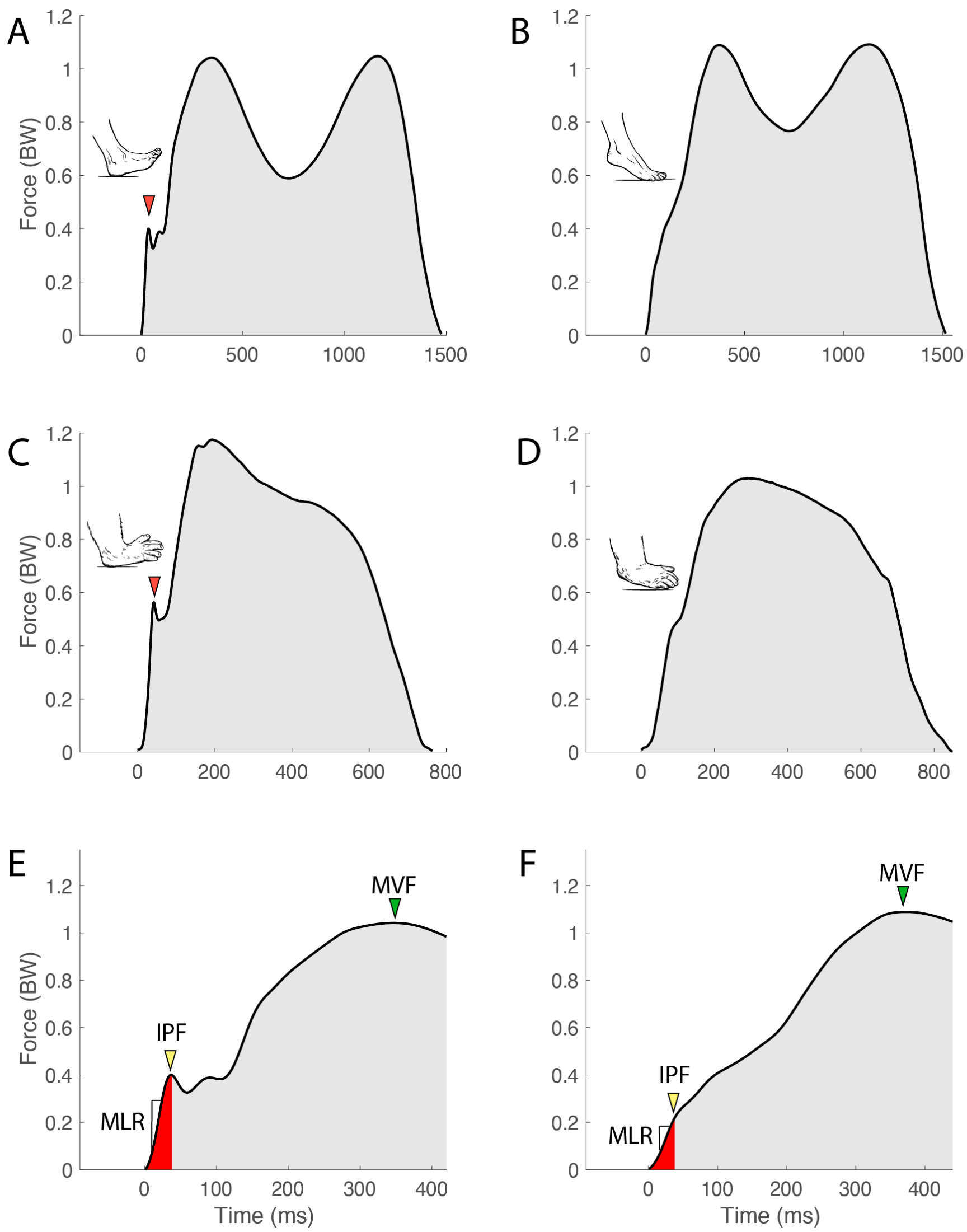

Fig. S1. Illustrative vertical ground reaction forces during stance phase of bipedal walking in humans (A, B, E, F) and chimpanzees (C, D). (A) and (C) depict heel-strike steps in humans and chimpanzees, respectively, with the red arrow indicating the impact force impulse. (B) and (D) depict midfoot-strike steps in humans and chimpanzees, respectively. (E) and (F) depict the force variables measured in this study in heel-strike and midfoot-strike steps, respectively: impact peak force (IPF), maximum vertical force (MVF), and maximum loading rate (MLR).

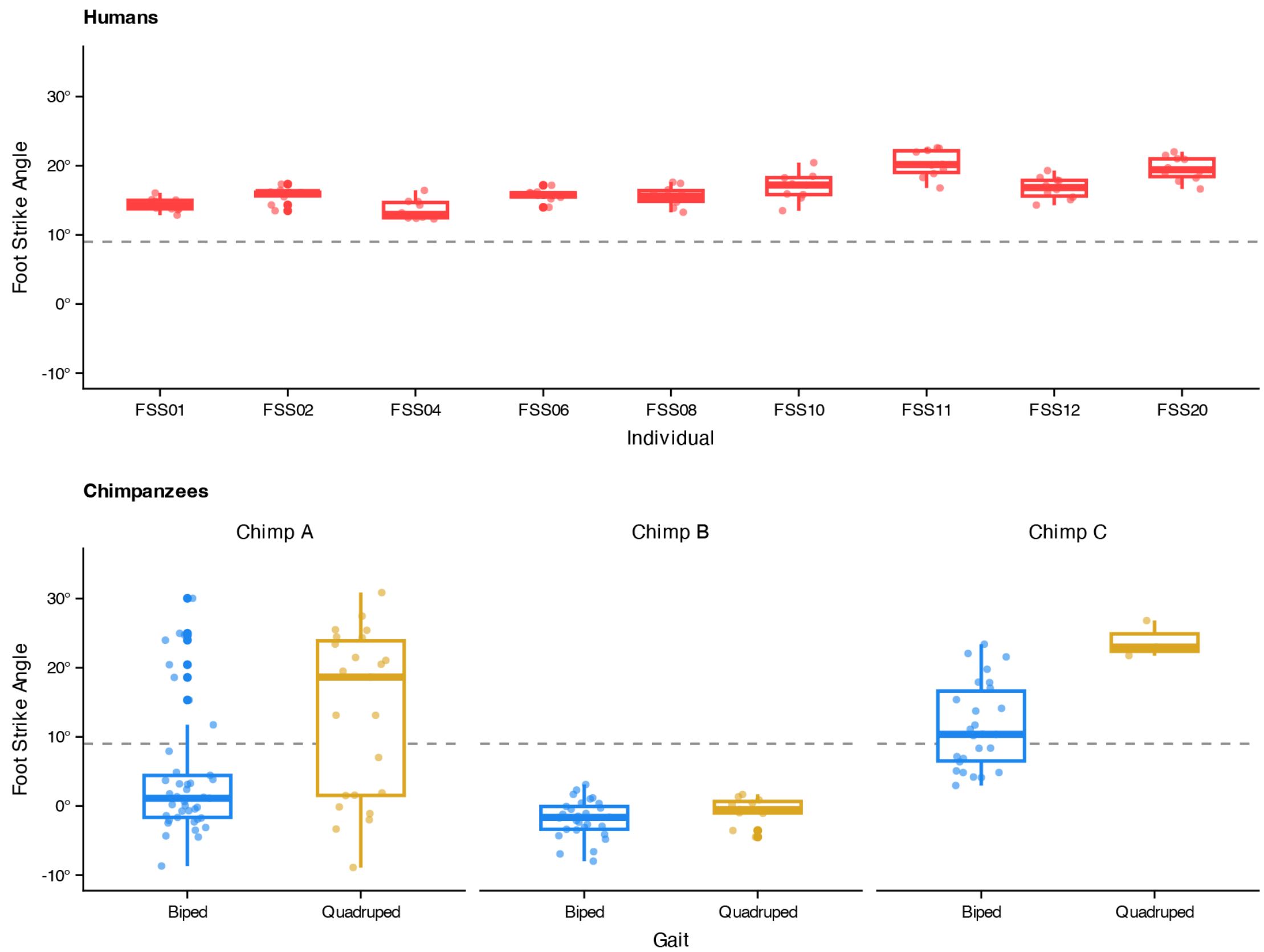

Fig. S2. Boxplots and points depicting raw data values of foot-strike angles (FSA) for human strides (n=89; 9.9 strides/subject), bipedal chimpanzee steps (Chimp A, n=44; Chimp B, n=31; Chimp C, n=26), and quadrupedal chimpanzee steps (Chimp A, n=32; Chimp B, n=12; Chimp C, n=3). In box plots, points represent individual steps, boxes represent interquartile ranges, middle bars represent median values, and whiskers extend to the most extreme data point ± 1.5 times the interquartile range. The gray dashed line indicates the approximate FSA threshold (9°) for different foot-strike types determined in this study. Steps above this line are heel-strikes, and steps below this line are midfoot-strikes.

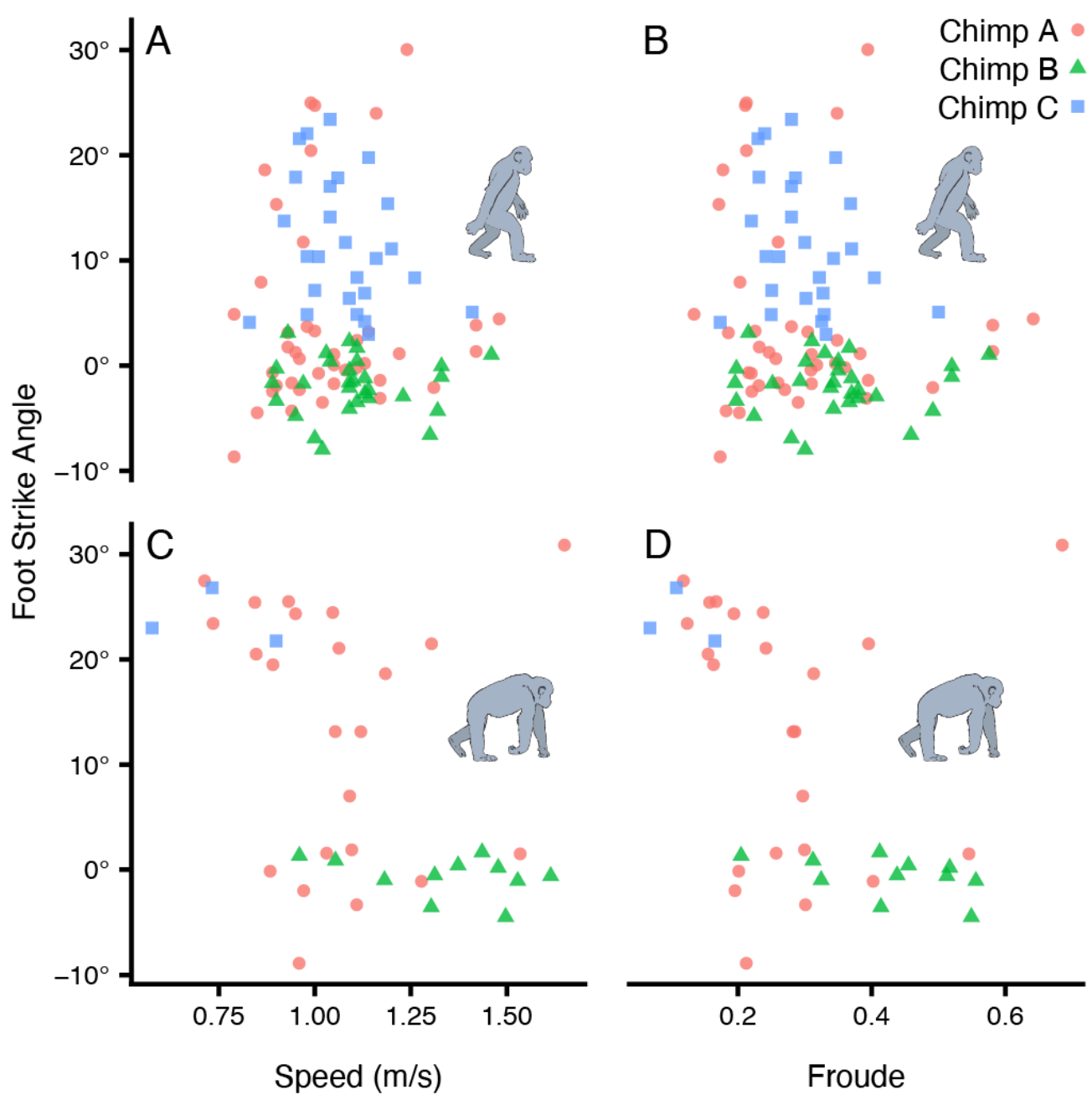

Fig. S3. Scatterplots indicating the absence of any relationship between walking speed and foot-strike angle (FSA) in chimpanzees. Datapoints are individual steps, with different colors for each chimpanzee subject. (A) and (B) depict bipedal steps (n=96), and (C) and (D) depict quadrupedal steps (n=37). (A) and (C) show absolute walking speed, and (B) and (D) show Froude number, which is a dimensionless measure of walking speed that accounts for the effects of differences in leg length on walking dynamics (8).

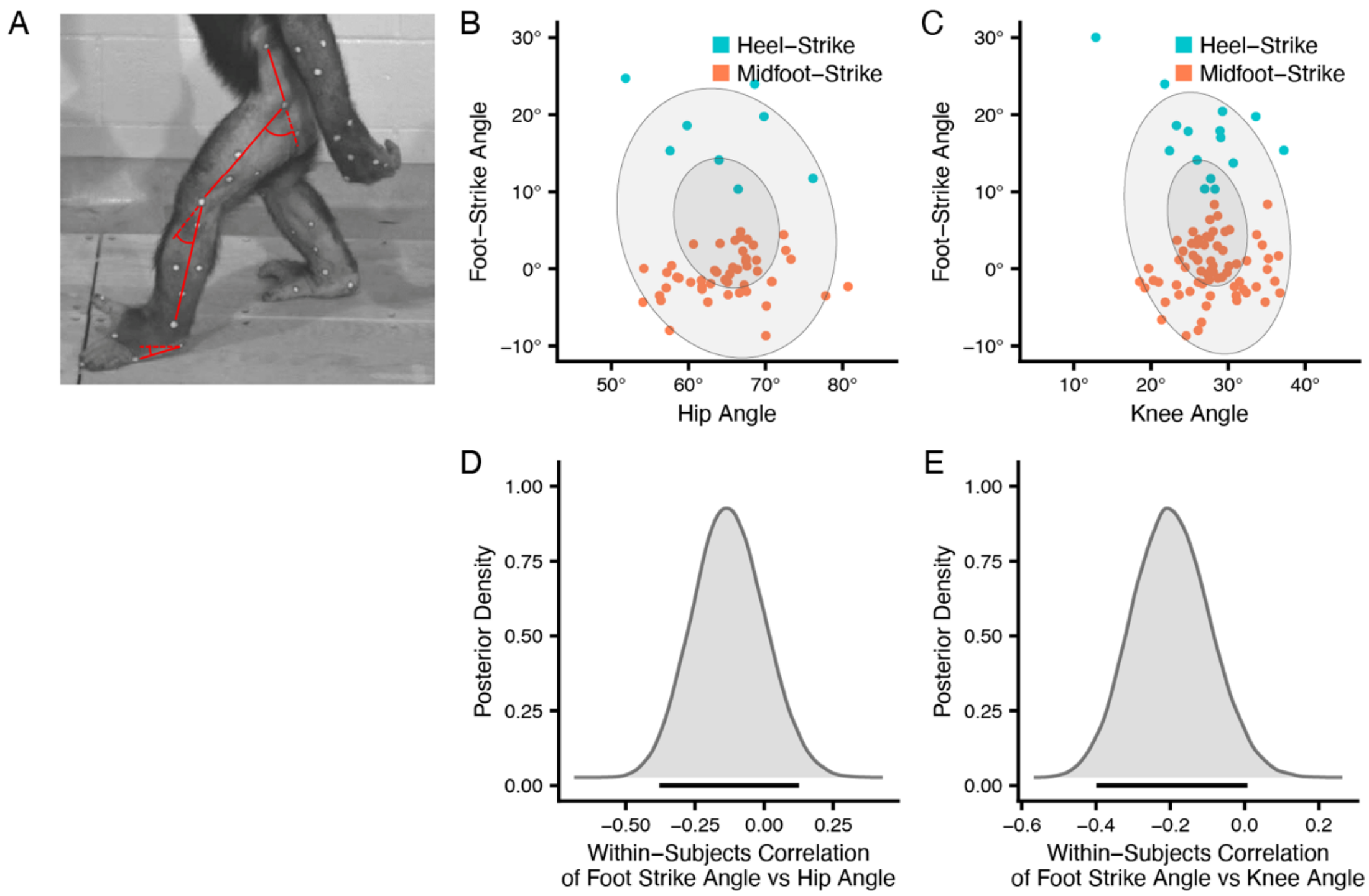

Fig. S4. Relationships between hip and knee angles at foot-strike and foot-strike angle (FSA). (A) Picture showing markers and segments used to quantify angles. (B) Scatterplot depicting FSA and hip angle at foot-strike for bipedal steps from this study (n=61). Polygon ellipses encompass 50% and 95% of the data. (C) Scatterplot depicting FSA and knee angle at foot-strike for bipedal steps from this study (n=88). Polygon ellipses encompass 50% and 95% of the data. (D) Posterior density plot depicting predicted distribution of within-subjects correlation coefficient between FSA and hip angle at foot-strike. Lower values indicate a stronger correlation. Solid line below plot indicates 95% HPDI. (E) Posterior density plot depicting predicted distribution of within-subjects correlation coefficient between FSA and knee angle at foot-strike. Lower values indicate a stronger correlation. Solid line below plot indicates 95% HPDI.

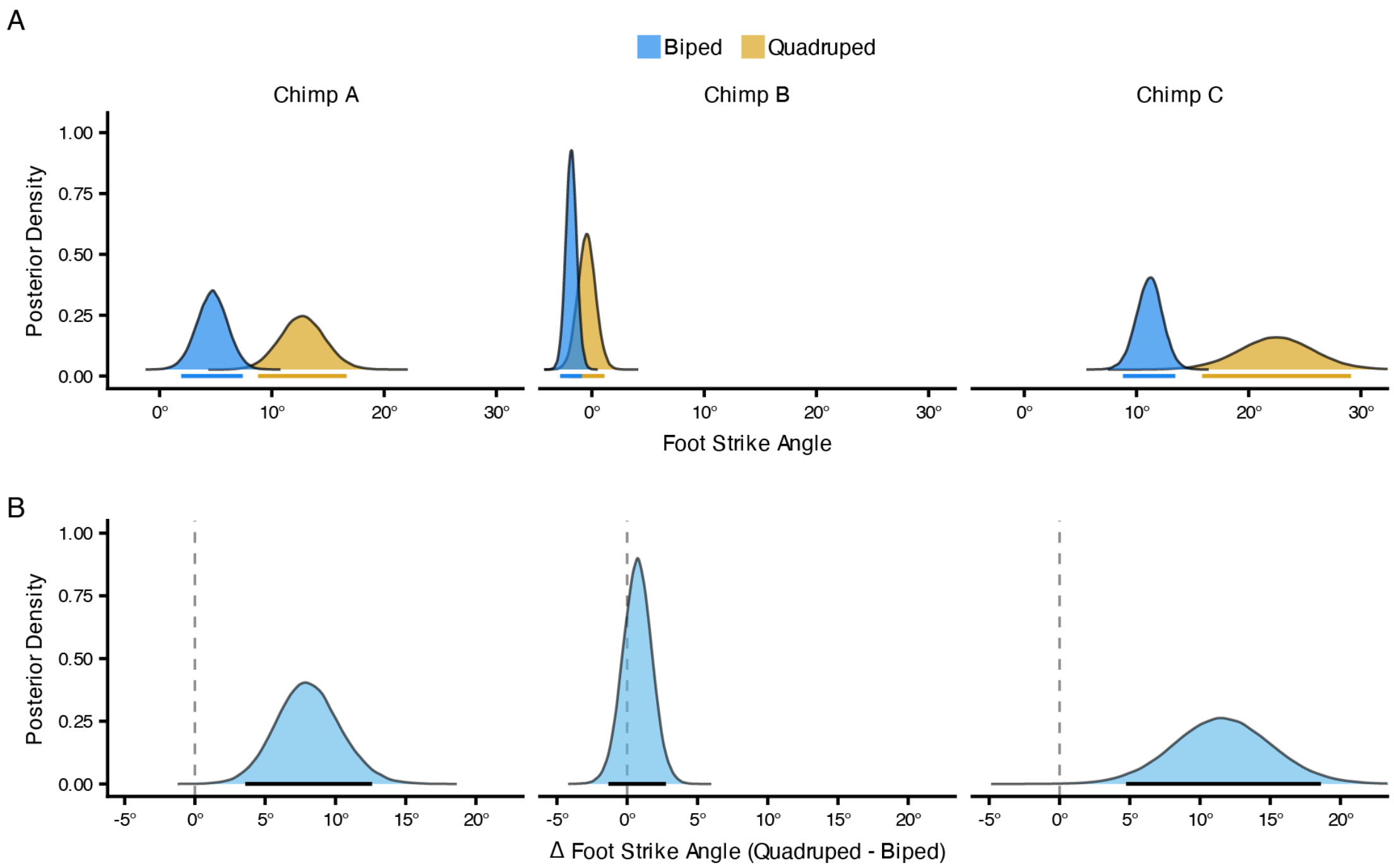

Fig. S5. Posterior density plots depicting Gaussian linear model-predicted distributions of foot-strike angle values for individual chimpanzee subjects. These plots demonstrate that all chimpanzee subjects in this study tended to use lower foot-strike angles, and thus more midfoot strikes, when bipedal than when quadrupedal. (A) Predicted distributions of geometric means of foot-strike angles for bipedal and quadrupedal steps. (B) Predicted distributions of arithmetic mean differences in foot-strike angle for quadrupedalism minus bipedalism for each individual chimpanzee subject. Vertical dashed line indicates 0°, with positive values indicating higher foot-strike angles in quadrupedalism. For (A) and (B), solid horizontal lines below distributions indicate 95% highest posterior density intervals.

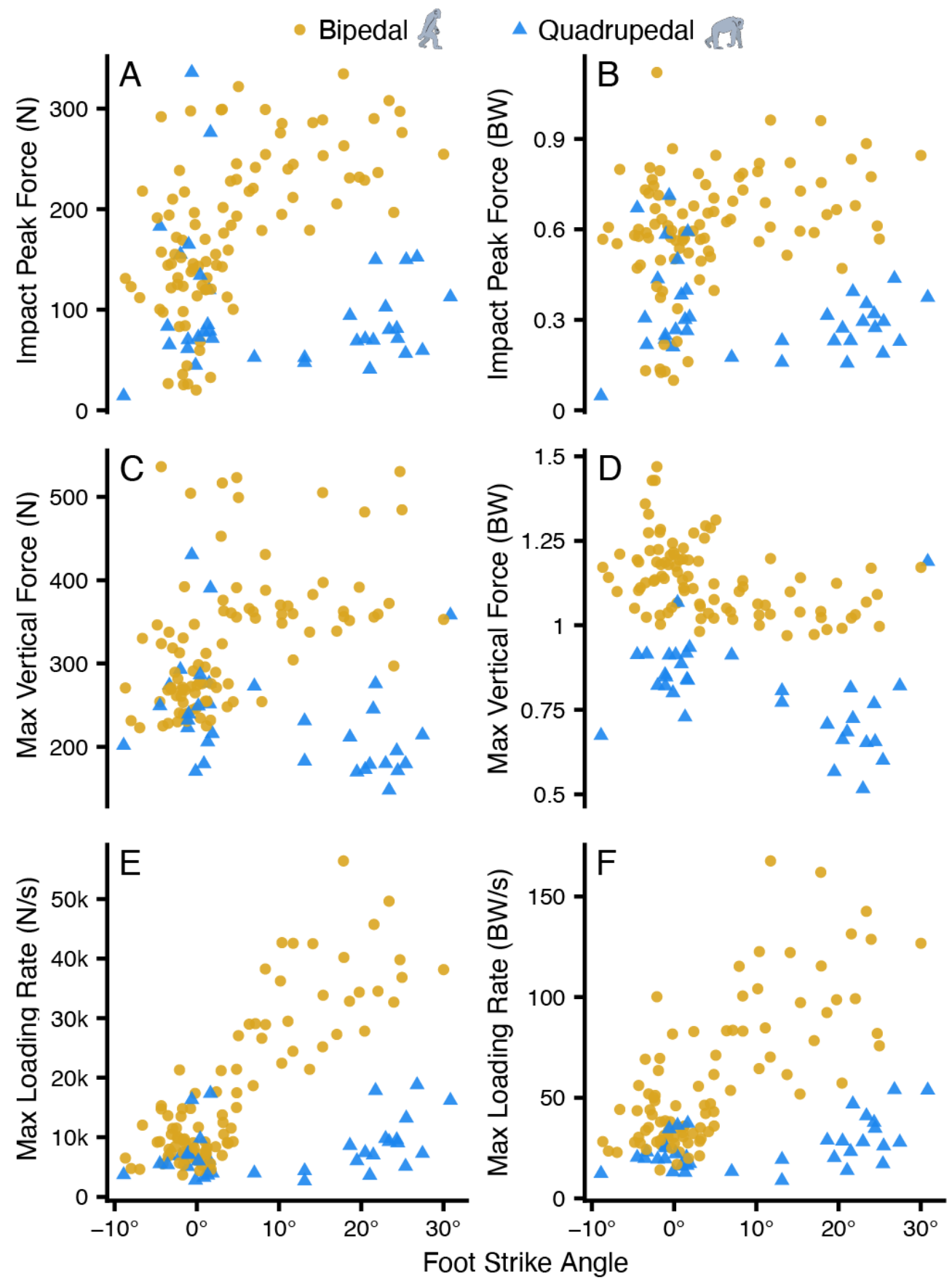

Fig. S6. Scatterplots depicting relationships between raw data values for foot-strike angle (FSA) and ground reaction force variables for bipedal (gold, n=101) and quadrupedal (blue, n=47) steps. (A, B) Impact peak force; (C, D) Maximum vertical force; (E, F) Maximum loading rate. (A, C, E) Force variables in absolute units of force (N). (B, D, F) Force variables in units of subject body weight (BW).

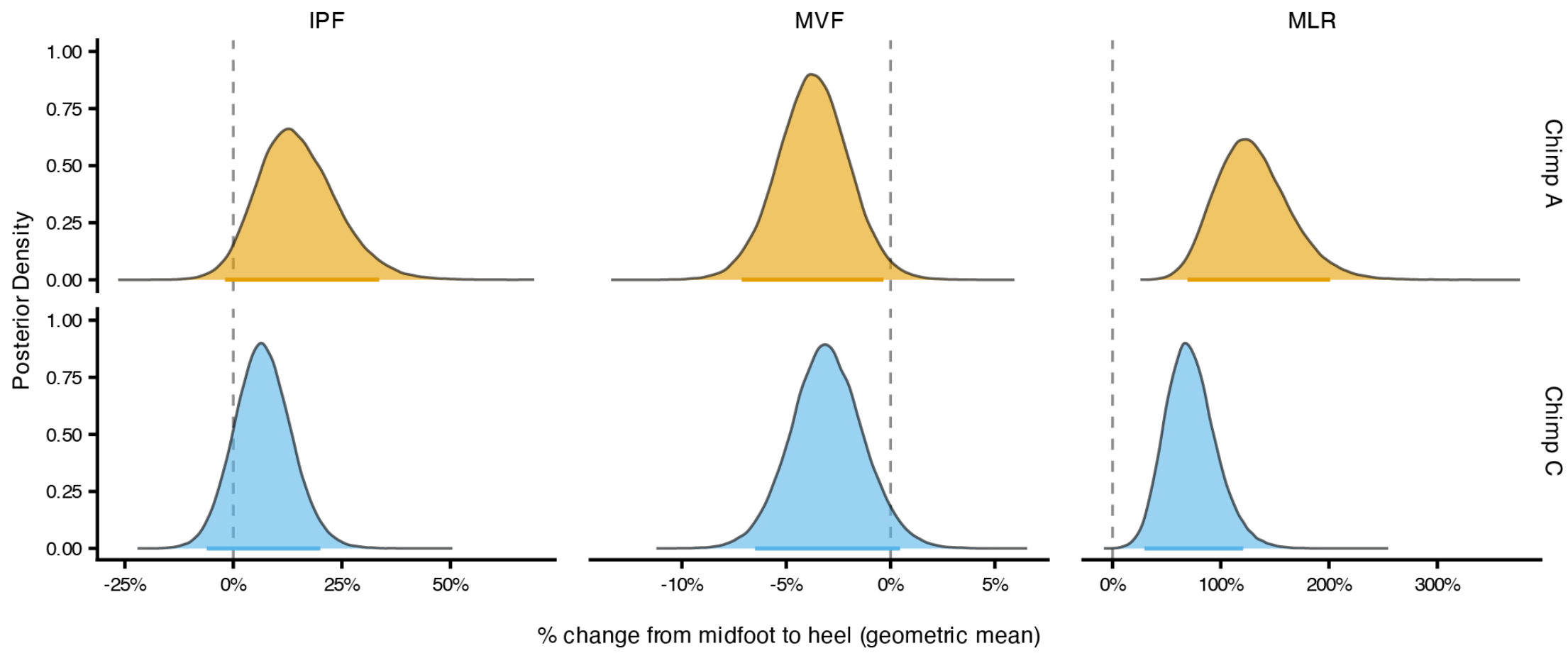

Fig. S7. Posterior density plots depicting the lognormal linear model-predicted distributions of percent change in the geometric means of impact peak force (IPF), maximum vertical force (MVF), and maximum loading rate (MLR) from midfoot-strike to heel-strike in bipedal walking for Chimp A (above) and Chimp C (below). Solid horizontal lines below distributions indicate 95% highest posterior density intervals**.** Dashed vertical lines indicate 0 (no difference), and higher percentages indicate higher values for heel-strikes.

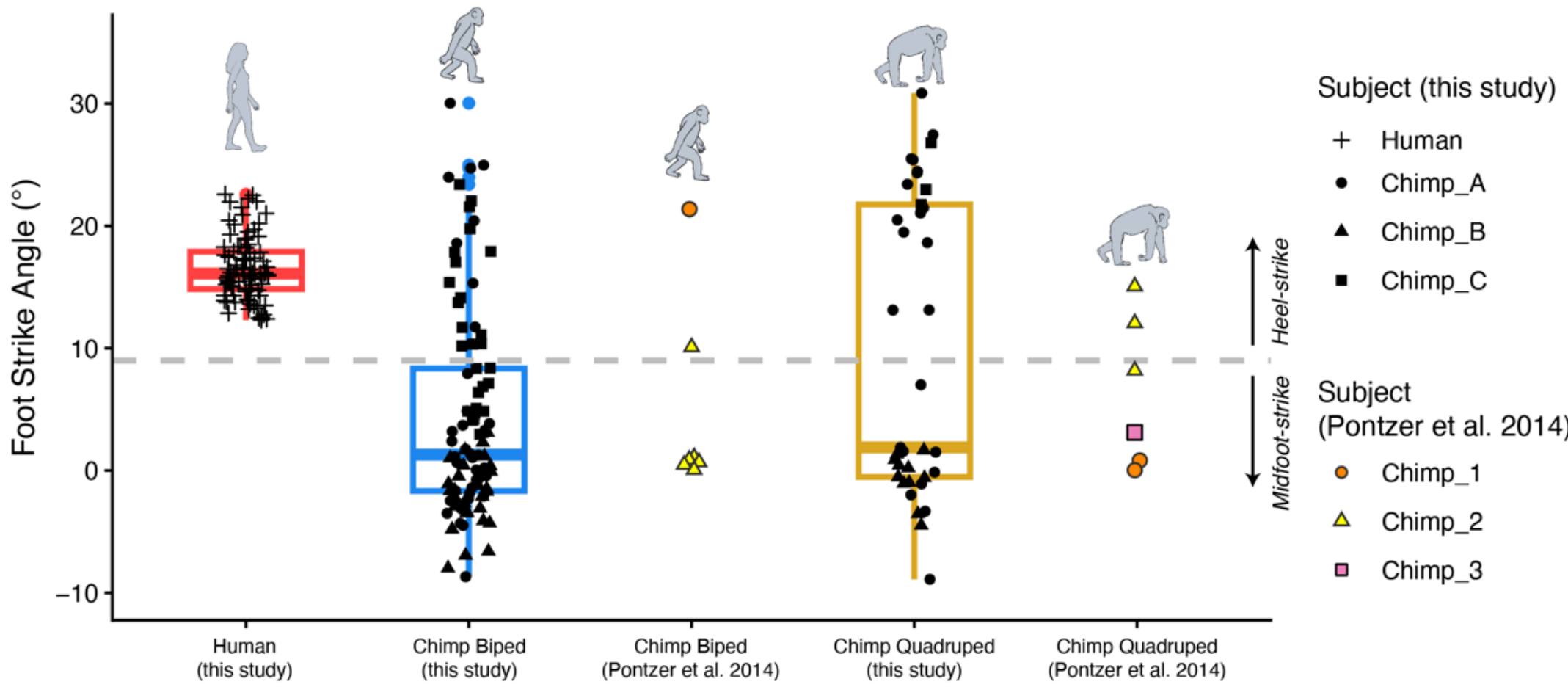

Fig. S8. Boxplots and points depicting raw data values of foot-strike angles (FSA) for human steps (this study, n=89), bipedal chimpanzee steps (this study, n=96, and Pontzer et al. (2014) (40), n=7), and quadrupedal chimpanzee steps (this study, n=37, and Pontzer et al. (2014) (40), n=6). In box plots, points represent individual steps, boxes represent interquartile ranges, middle bars represent median values, and whiskers extend to the most extreme data point ± 1.5 times the interquartile range. Different symbols/colors for datapoints represent steps from different subjects. The gray dashed line indicates the approximate FSA threshold (9°) for different foot-strike types determined in this study. Steps above this line are heel-strikes, and steps below this line are midfoot-strikes.

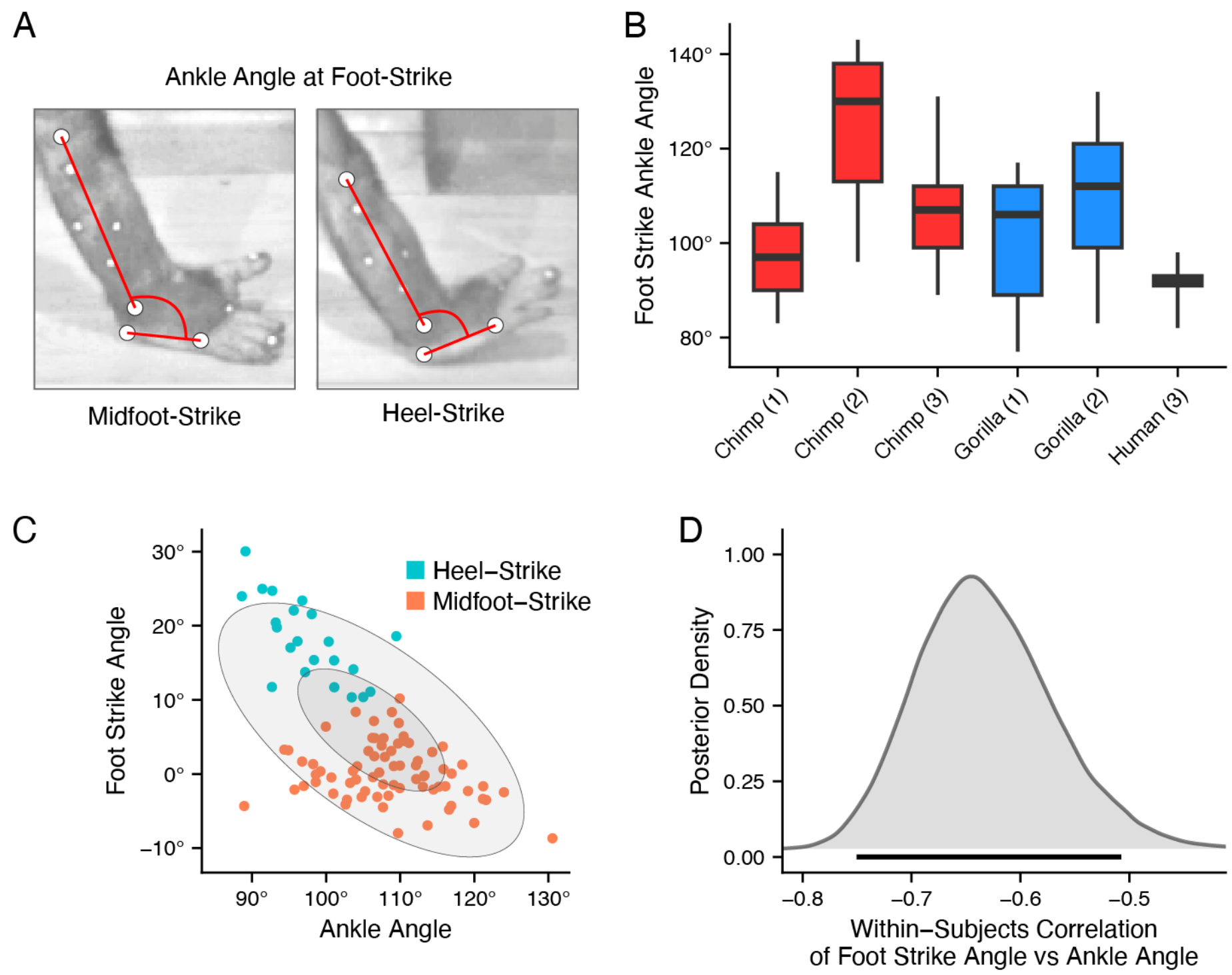

Fig. S9. Relationship between ankle angle at foot-strike and foot-strike angle (FSA). (A) Pictures showing markers and segments used to quantify ankle angle. (B) Foot-strike (FS) ankle angles from [1] Finestone et al. 2018 (41), [2] Zeininger et al. 2020 (13), and [3] this study. Boxes represent interquartile ranges, middle bars represent median values, and whiskers extend to the most extreme data point ± 1.5 times the interquartile range. Data in boxplots labeled with [1] and [2] are re-drawn from data depicted in Fig. 5B in (41) and Fig. 4C in (13), respectively. (C) Scatterplot depicting FSA and ankle angle at foot-strike for steps from this study (n=97). Polygon ellipses encompass 50% and 95% of the data. (D) Posterior density plot depicting predicted distribution of within-subjects correlation coefficient between FSA and ankle angle at foot-strike. Lower values indicate a stronger correlation. Solid line below plot indicates 95% HPDI.

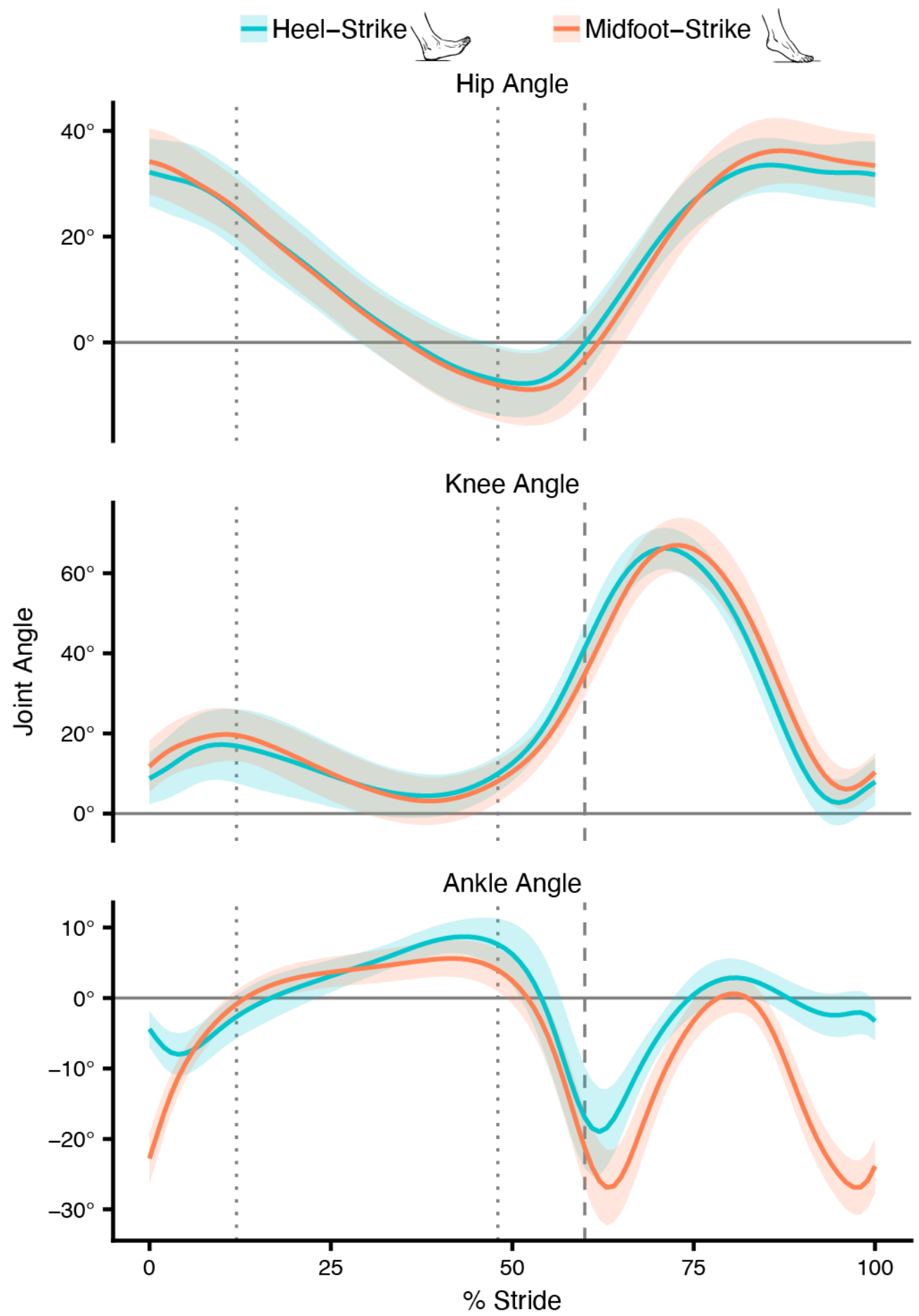

Fig. S10. Kinematic waveform graphs depicting average hip, knee, and ankle angles across a stride in human subjects walking with heel-strikes and midfoot-strikes. Solid green lines represent averages of all heel-strikes (n=110), and solid orange lines represent averages of all midfoot-strikes (n=110). Shaded regions represent ± one standard deviation at each percentage of a stride. Area between dotted lines is single support period of stance phase, and dashed line indicates time of transition between stance and swing phase. Positive hip and knee angles indicate flexion, and positive ankle angles indicate dorsiflexion.

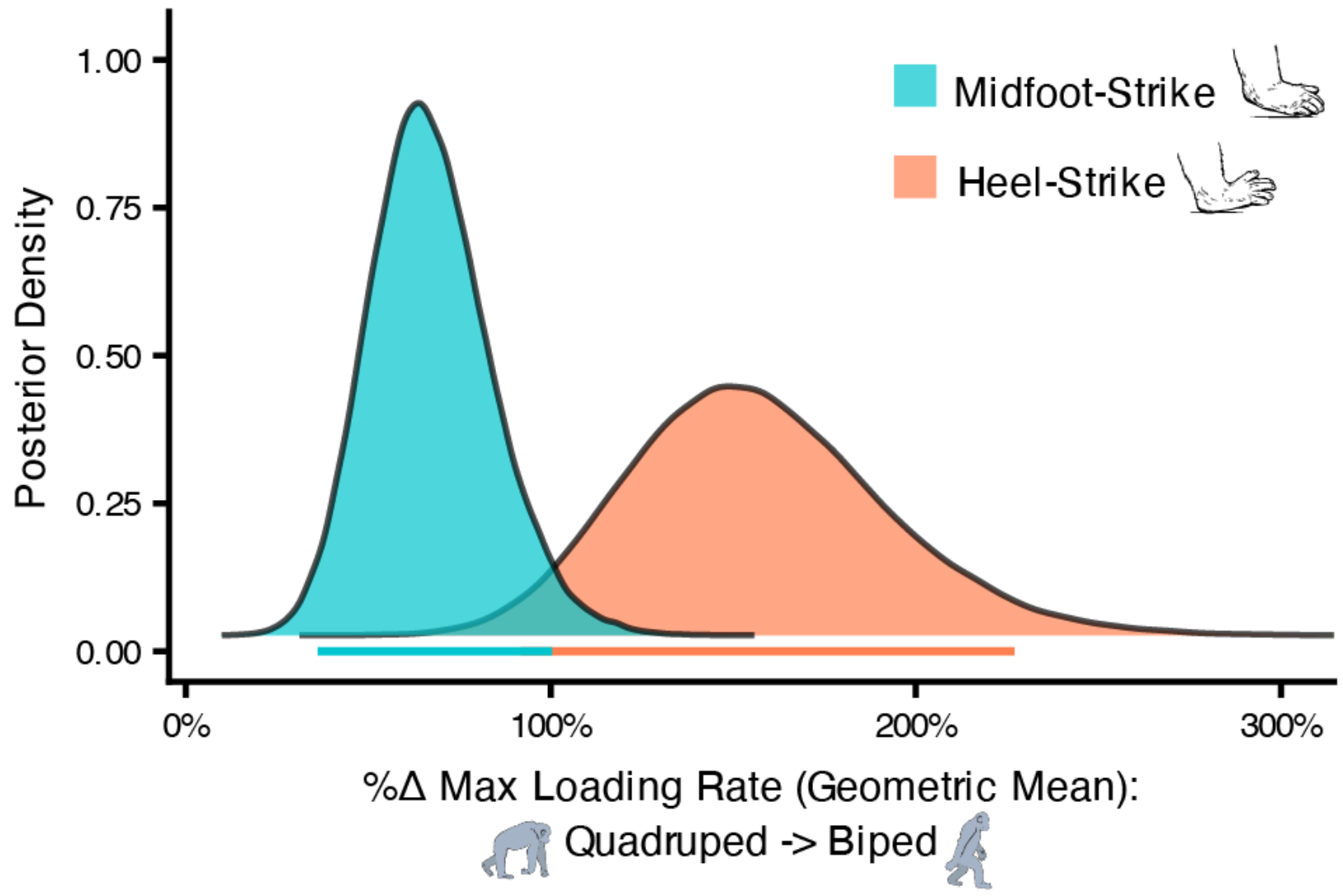

Fig. S11. Posterior density plot depicting lognormal linear model-predicted distributions of percent change (Δ%) in the geometric means of MLR from quadrupedal to bipedal steps in chimpanzees walking with midfoot-strikes (green), and heel-strikes (orange). Bars below plots indicate 95% highest posterior density intervals, which were 36.2 to 100.4% for midfoot-strikes, and 91.7 to 227% for heel-strikes.

Tables

Table S1. Chimpanzee subject information. Number of bipedal (BP) and quadrupedal (QP) steps included in analysis is listed. Use of kinematic ('Kin') data to calculated foot-strike angle is indicated. Use of ground reaction force (GRF) data to calculate impact peak force, maximum vertical force, and maximum loading rate is indicated.

Subject ID	Sex	Age (years)	Mass (kg)	BP steps (n)	QP steps (n)	Kin data	GRF data
A[1]	Male	4.7–8.3	20.1–51.8	44	32	Yes	Yes
B[1]	Male	5.4–8.8	20.7–48.1	31	12	Yes	Yes
C[1]	Male	5.8–5.9	35.5–38.8	26	3	Yes	Yes
1[2]	Male	6	34.2	1	2	Yes	No
2[2]	Female	19	61.8	6	3	Yes	No
3[2]	Male	9	52.8	0	1	Yes	No

[1]Data from present study. Data were collected over the course of ~4 years.
[2]Data from Pontzer et al. (40).

Table S2. Human subject information. 'GRF study' indicates subjects who participated in data collection sessions where ground reaction force (GRF) and foot-strike angle data were collected while subjects walked at a range of speeds (see Figs. 1 and 2). 'Metabolic study' indicates subjects who participated in data collection sessions where metabolic data and kinematic data were collected while subjects walked with different foot-strike postures at matched speeds (see Figs. 3 and S7, Table S3).

Subject	Sex	Age	Mass[1]	FSA study	Metabolic study
FSS01	M	36	65.4 (64.3)	Yes	Yes
FSS02	M	25	86.4 (91.1)	Yes	Yes
FSS04	M	20	76.2 (80.1)	Yes	Yes
FSS06	F	24	58.3	Yes	No
FSS08	M	28	69.7 (71.4)	Yes	Yes
FSS10	F	31	61.5 (60.1)	Yes	Yes
FSS11	F	32	80.9 (79.1)	Yes	Yes
FSS12	F	20	67.3 (69.2)	Yes	Yes
FSS20	F	32	48.9 (48.1)	Yes	Yes
FSS24	M	27	(82.1)	No	Yes
FSS25	F	24	(93.1)	No	Yes
FSS26	M	24	(87.1)	No	Yes

[1]Numbers outside parentheses indicate subject mass during FSA study, numbers inside parentheses indicate subject mass during Metabolic study.

Table S3. Spatiotemporal walking kinematics for human subjects walking with heel-strikes (HS) and midfoot-strikes (MS) at matched dimensionless speeds (Froude = 0.18, speed = 1.25±0.03 m/s).

	Stride Length (m)		Stride Time (s)		Stride Frequency (s^{-1})		Stance Time (s)		Swing Time (s)		Duty Factor	
**Subject**	**HS**	**MS**	**HS**	**MS**	**HS**	**MS**	**HS**	**MS**	**HS**	**MS**	**HS**	**MS**
**FSS01**	1.31	1.3	1.03	1.03	0.968	0.968	0.628	0.636	0.406	0.397	0.607	0.615
**FSS02**	1.29	1.31	1.02	1.04	0.983	0.959	0.648	0.666	0.369	0.377	0.637	0.639
**FSS04**	1.29	1.27	1.05	1.02	0.952	0.978	0.639	0.634	0.413	0.389	0.607	0.62
**FSS08**	1.25	1.23	1.04	1.01	0.964	0.99	0.612	0.631	0.427	0.379	0.589	0.625
**FSS10**	1.28	1.25	1.05	1.02	0.95	0.982	0.63	0.626	0.423	0.393	0.598	0.615
**FSS11**	1.23	1.18	1.04	1	0.965	0.998	0.632	0.613	0.405	0.389	0.61	0.612
**FSS12**	1.27	1.27	1.03	1.01	0.968	0.994	0.613	0.613	0.421	0.393	0.593	0.609
**FSS20**	1.4	1.29	1.12	1.03	0.896	0.968	0.642	0.619	0.475	0.415	0.575	0.599
**FSS24**	1.26	1.26	1.03	1	0.973	0.998	0.631	0.63	0.397	0.372	0.614	0.629
**FSS25**	1.27	1.27	1.03	1.03	0.973	0.973	0.639	0.655	0.391	0.375	0.621	0.636
**FSS26**	1.3	1.32	1.07	1.08	0.936	0.929	0.661	0.666	0.408	0.411	0.618	0.619
**Average**	1.29	1.27	1.05	1.02	0.957	0.976	0.634	0.635	0.412	0.390	0.606	0.620

Table S4. Comparison of average percent metabolic cost increases observed in a selection of studies where human subjects altered some feature of their normal, preferred walking (or running) kinematics as part of an experimental intervention. Percentages are calculated as metabolic cost (cost of transport or cost of locomotion) measured during kinematic adjustment condition divided by unadjusted condition times one hundred percent. These values are reported in the cited article, calculated from reported results, or extracted from article figures where indicated. In all cases, walking/running speed was the same in both conditions. This table is not intended to be comprehensive of all studies investigating the effects of altered walking kinematics on human metabolic energy expenditure. Rather, it provides examples that give context for the magnitude of cost difference between heel-strike and midfoot-strike walking measured in the present study.

Kinematic adjustment	Description of Kinematic Adjustment	Average % Metabolic Cost Increase	Source
Midfoot-strikes (walking)	During walking, subjects contacted ground with the lateral edge of the foot near the metatarsal heads before bringing the heel down.	33.6%	Present study
Midfoot-strikes (running)	Subjects who typically ran with heel-strikes ran with midfoot-strikes (called 'forefoot strikes' in this article), which involved landing with an ankle dorsiflexion angle of <5° and a center of pressure position beyond 30% of foot length.	5.5%	Gruber et al. 2013 (42)
Enforced step width	Subjects were required to walk with step widths that were a specific proportion of their leg length *L*. They also walked using their preferred step widths. Subjects were estimated to use 14% higher costs of transport when required to maintain step widths near their preferred width than they used when walking freely without trying to maintain a specific step width.	14%	Donelan et al. 2001 (43)
Double preferred step width	See above for study description. Subjects walked with an enforced step width near their preferred width (0.15*L*) and an enforced step width double that (0.3*L*). Subjects had 16% higher costs of transport in the latter condition compared to the former condition.	16%[1]	Donelan et al. 2001 (43)
-15% reduced step frequency	Subjects walked at their preferred step frequency, which was recorded by the study team. Subjects then walked matching their footfalls to a metronome that was set at 5% intervals above and below their preferred frequency. When they walked with frequencies 15% below their preferred frequency, which should correspond to spatially longer than preferred strides, they had 17% higher costs of transport than when walking at their preferred frequency.	17%[1]	Selinger et al. 2017 (44)
+15% increased step frequency	See above for study description. Here subjects walked with a step frequency that was 15% above their preferred frequency. This should correspond to spatially shorter than preferred strides.	15%[1]	Selinger et al. 2017 (44)

9x increase lateral leg swing	Subjects walked with lightweight, medially-oriented “fins” attached to their shanks that required them to circumduct their lower limbs more than normal during swing phase. In this condition, the fins caused participants to increase the lateral deviation of their swing lower limb such that it was 9 times greater than the deviation that occurs during normal walking without a fin (but with appropriate weight adjustment).	22%	Shorter et al. 2017 (45)
No arm swing	Subjects walked while holding their upper limbs at the sides of their bodies, rather than allowing them to swing naturally.	12%[1]	Collins et al. 2009 (46)
Anti-normal arm swing	Subjects walked while swinging their upper limbs forward and backward in tandem with the ipsilateral lower limb.	26%[1]	Collins et al. 2009 (46)

[1]Value calculated based on average results extracted from article figure.

Movies

Movie S1 (separate file). Chimpanzee A walking quadrupedally with heel- and midfoot-strikes.

Movie S2 (separate file). Chimpanzee A walking bipedally with heel- and midfoot-strikes.

Movie S3 (separate file). Chimpanzee B walking quadrupedally with midfoot-strikes.

Movie S4 (separate file). Chimpanzee B walking bipedally with midfoot-strikes.

Movie S5 (separate file). Chimpanzee C walking quadrupedally with heel-strikes.

Movie S6 (separate file). Chimpanzee C walking bipedally with heel- and midfoot-strikes.

Movie S7 (separate file). Human subject walking on treadmill with heel-strikes.

Movie S8 (separate file). Human subject walking on treadmill with midfoot-strikes.

Dataset S1 (separate file). Processed kinematic and kinetic data from chimpanzee and human subjects used in main analyses (Figs. 1-3, S2-6).

SI References

1. D. L. Gebo, Plantigrady and foot adaptation in African apes: Implications for hominid origins. *American Journal of Physical Anthropology* **89**, 29–58 (1992).
2. D. L. Gebo, Reply to Dr. Meldrum. *American Journal of Physical Anthropology* **91**, 382–385 (1993).
3. D. J. Meldrum, On plantigrady and quadrupedalism. *American Journal of Physical Anthropology* **91**, 379–381 (1993).
4. D. Schmitt, S. G. Larson, Heel contact as a function of substrate type and speed in primates. *American Journal of Physical Anthropology* **96**, 39–50 (1995).
5. H. Pontzer, Predicting the energy cost of terrestrial locomotion: a test of the LiMb model in humans and quadrupeds. *Journal of Experimental Biology* **210**, 484–494 (2007).
6. H. Pontzer, A new model predicting locomotor cost from limb length *via* force production. *Journal of Experimental Biology* **208**, 1513–1524 (2005).
7. J. T. Webber, D. A. Raichlen, The role of plantigrady and heel-strike in the mechanics and energetics of human walking with implications for the evolution of the human foot. *Journal of Experimental Biology* **219**, 3729–3737 (2016).
8. R. McN. Alexander, A. S. Jayes, A dynamic similarity hypothesis for the gaits of quadrupedal mammals. *Journal of Zoology* **201**, 135–152 (1983).
9. A. A. Biewener, C. T. Farley, T. J. Roberts, M. Temaner, Muscle mechanical advantage of human walking and running: implications for energy cost. *Journal of Applied Physiology* **97**, 2266–2274 (2004).
10. J. DeSilva, E. McNutt, J. Benoit, B. Zipfel, One small step: A review of Plio-Pleistocene hominin foot evolution. *American Journal of Physical Anthropology* **168**, 63–140 (2019).
11. K. G. Hatala, S. M. Gatesy, P. L. Falkingham, Arched footprints preserve the motions of fossil hominin feet. *Nature Ecology and Evolution* **7**, 32–41 (2023).
12. T. C. Prang, The relative size of the calcaneal tuber reflects heel strike plantigrady in African apes and humans. *American Journal of Biological Anthropology* **183**, e24865 (2024).

13. A. Zeininger, D. Schmitt, R. E. Wunderlich, Mechanics of heel-strike plantigrady in African apes. *Journal of Human Evolution* **145**, 102840 (2020).
14. R. M. Mesquita, G. Catavitello, P. A. Willems, A. H. Dewolf, Modification of the locomotor pattern when deviating from the characteristic heel-to-toe rolling pattern during walking. *European Journal of Applied Physiology* **123**, 1455–1467 (2023).
15. R. Kram, C. R. Taylor, Energetics of running: a new perspective. *Nature* **346**, 265–267 (1990).
16. T. McGeer, Passive dynamic walking. *International Journal of Robotics Research* **9**, 62–82 (1990).
17. A. H. Hansen, D. S. Childress, E. H. Knox, Roll-over shapes of human locomotor systems: effects of walking speed. *Clinical Biomechanics* **19**, 407–414 (2004).
18. P. G. Adamczyk, S. H. Collins, A. D. Kuo, The advantages of a rolling foot in human walking. *Journal of Experimental Biology* **209**, 3953–3963 (2006).
19. A. Ruina, J. E. A. Bertram, M. Srinivasan, A collisional model of the energetic cost of support work qualitatively explains leg sequencing in walking and galloping, pseudo-elastic leg behavior in running and the walk-to-run transition. *Journal of Theoretical Biology* **237**, 170–192 (2005).
20. J. L. Croft, J. E. A. Bertram, Form in the context of function: Fundamentals of an energy effective striding walk, the role of the plantigrade foot and its expected size. *American Journal of Physical Anthropology* **173**, 760–767 (2020).
21. M. Kwan, M. Hubbard, Optimal foot shape for a passive dynamic biped. *Journal of Theoretical Biology* **248**, 331–339 (2007).
22. A. D. Kuo, J. M. Donelan, A. Ruina, Energetic Consequences of Walking Like an Inverted Pendulum: Step-to-Step Transitions: *Exercise and Sport Sciences Reviews* **33**, 88–97 (2005).
23. P. G. Adamczyk, A. D. Kuo, Mechanical and energetic consequences of rolling foot shape in human walking. *Journal of Experimental Biology* jeb.082347 (2013). https://doi.org/10.1242/jeb.082347.
24. K. E. Zelik, A. D. Kuo, Human walking isn't all hard work: evidence of soft tissue contributions to energy dissipation and return. *Journal of Experimental Biology* **213**, 4257–4264 (2010).
25. K. E. Zelik, K. Z. Takahashi, G. S. Sawicki, Six degree-of-freedom analysis of hip, knee, ankle and foot provides updated understanding of biomechanical work during human walking. *Journal of Experimental Biology* **218**, 876–886 (2015).
26. T. J. Van Der Zee, A. D. Kuo, Soft tissue deformations explain most of the mechanical work variations of human walking. *Journal of Experimental Biology* **224**, jeb239889 (2021).
27. U. Jørgensen, Achillodynia and loss of heel pad shock absorbency. *American Journal of Sports Medicine* **13**, 128–132 (1985).
28. P. Aerts, R. F. Ker, D. De Clercq, D. W. Ilsley, R. McN. Alexander, The mechanical properties of the human heel pad: A paradox resolved. *Journal of Biomechanics* **28**, 1299–1308 (1995).
29. M. T. G. Pain, J. H. Challis, The role of the heel pad and shank soft tissue during impacts: a further resolution of a paradox. *Journal of Biomechanics* **34**, 327–333 (2001).
30. A. Gefen, M. Megido-Ravid, Y. Itzchak, In vivo biomechanical behavior of the human heel pad during the stance phase of gait. *Journal of Biomechanics* **34**, 1661–1665 (2001).
31. P. M. Baines, A. L. Schwab, A. J. Van Soest, Experimental estimation of energy absorption during heel strike in human barefoot walking. *PLoS ONE* **13**, e0197428 (2018).
32. J. R. Usherwood, A. J. Channon, J. P. Myatt, J. W. Rankin, T. Y. Hubel, The human foot and heel–sole–toe walking strategy: a mechanism enabling an inverted pendular gait with low isometric muscle force? *Journal of the Royal Society Interface.* **9**, 2396–2402 (2012).
33. N. B. Holowka, M. C. O'Neill, Three-dimensional moment arms and architecture of chimpanzee (*Pan troglodytes*) leg musculature. *Journal of Anatomy* **223**, 610–628 (2013).
34. J. P. Myatt, R. H. Crompton, S. K. S. Thorpe, Hindlimb muscle architecture in non-human great apes and a comparison of methods for analysing inter-species variation: Comparing hindlimb muscle architecture in non-human great apes. *Journal of Anatomy* **219**, 150–166 (2011).

35. S. K. S. Thorpe, R. H. Crompton, M. M. Günther, R. F. Ker, R. McNeill Alexander, Dimensions and moment arms of the hind- and forelimb muscles of common chimpanzees (Pan troglodytes). *American Journal of Physical Anthropology* **110**, 179–199 (1999).
36. G. G. Handsfield, C. H. Meyer, J. M. Hart, M. F. Abel, S. S. Blemker, Relationships of 35 lower limb muscles to height and body mass quantified using MRI. *Journal of Biomechanics* **47**, 631–638 (2014).
37. C. W. Spoor, J. L. Van Leeuwen, C. G. M. Meskers, A. F. Titulaer, A. Huson, Estimation of instantaneous moment arms of lower-leg muscles. *Journal of Biomechanics* **23**, 1247–1259 (1990).
38. R. C. Payne, *et al.*, Morphological analysis of the hindlimb in apes and humans. II. Moment arms. *Journal of Anatomy* **208**, 725–742 (2006).
39. M. C. O'Neill, A. Nagano, B. R. Umberger, A three-dimensional musculoskeletal model of the pelvis and lower limb of *Australopithecus afarensis*. *American Journal of Biological Anthropology* **183**, e24845 (2024).
40. H. Pontzer, D. A. Raichlen, P. S. Rodman, Bipedal and quadrupedal locomotion in chimpanzees. *Journal of Human Evolution* **66**, 64–82 (2014).
41. E. M. Finestone, M. H. Brown, S. R. Ross, H. Pontzer, Great ape walking kinematics: Implications for hominoid evolution. *American Journal of Physical Anthropology* **166**, 43–55 (2018).
42. A. H. Gruber, B. R. Umberger, B. Braun, J. Hamill, Economy and rate of carbohydrate oxidation during running with rearfoot and forefoot strike patterns. *Journal of Applied Physiology* **115**, 194–201 (2013).
43. J. M. Donelan, R. Kram, K. Arthur D., Mechanical and metabolic determinants of the preferred step width in human walking. *Proceeding of the Royal Society B* **268**, 1985–1992 (2001).
44. J. C. Selinger, S. M. O'Connor, J. D. Wong, J. M. Donelan, Humans can continuously optimize energetic cost during walking. *Current Biology* **25**, 2452–2456 (2015).
45. K. A. Shorter, A. Wu, A. D. Kuo, The high cost of swing leg circumduction during human walking. *Gait & Posture* **54**, 265–270 (2017).
46. S. H. Collins, P. G. Adamczyk, A. D. Kuo, Dynamic arm swinging in human walking. *Proceeding of the Royal Society B* **276**, 3679–3688 (2009).
